# A multidimensional analysis of temporomandibular joint and ankle joint erosion in inflammatory arthritis

**DOI:** 10.3389/fimmu.2025.1560723

**Published:** 2025-07-18

**Authors:** Darja Andreev, Pauline Porschitz, Daniela Weidner, Rui Song, Matthias Weider, Georg Schett, Lina Gölz, Aline Bozec

**Affiliations:** ^1^ Center for Regenerative Therapies Dresden (CRTD), Technische Universität (TU) Dresden, Dresden, Germany; ^2^ Department of Medicine 3 - Rheumatology and Immunology, Friedrich-Alexander-Universität Erlangen-Nürnberg (FAU) and Universitätsklinikum Erlangen (UKER), Erlangen, Germany; ^3^ Deutsches Zentrum Immuntherapie (DZI), FAU and UKER, Erlangen, Germany; ^4^ Exploratory Research Unit, Optical Imaging Centre Erlangen, FAU, Erlangen, Germany; ^5^ Dental Clinic 3 – Department of Orthodontics and Orofacial Orthopedics, FAU and UKER, Erlangen, Germany

**Keywords:** inflammatory arthritis, rheumatoid arthritis, juvenile idiopathic arthritis temporomandibular joint, temporomandibular disorder, human TNF-α transgenic mice, bone erosion, osteoclasts

## Abstract

Rheumatoid arthritis (RA) and other inflammatory arthritis are systemic diseases that primarily affect the joints, characterized by synovial inflammation and progressive cartilage and bone degradation. The temporomandibular joint (TMJ) is reported to be involved in over 50% of RA cases, often leading to severe jaw pain and compromised oral function. Despite its prevalence, TMJ involvement is often underestimated, and its cellular and molecular mechanisms remain poorly understood. Due to the unique biological and functional properties of the TMJ, inflammatory pathways observed in other joints such as the well-studied ankle joint may not directly apply to the TMJ. This study aimed to establish a reliable inflammatory arthritis model for investigating TMJ-specific pathomechanisms. The human TNF-α transgenic (hTNFtg) mouse model effectively replicated TMJ pathology seen in arthritic patients, including increased synovial inflammation (*p*=0.0024) and severe bone loss (*p*=0.009) as compared to control mice assessed by micro-computed tomography and histomorphometry. These changes were driven by increased osteoclast numbers (*p*=0.0331) and upregulation of genes associated with bone resorption such as *Acp5* (*p*=0.0003) and *Ctsk* (*p*=0.0025). Notably, we observed that the TMJ displays a unique pattern of immune cell infiltration and pro-inflammatory cytokine expression compared to the ankle joint, particularly with respect to T cell recruitment. These findings were further supported by bulk RNA sequencing, which revealed overall increased inflammation in both the ankle joint and TMJ of hTNFtg mice compared to the control group. Interestingly, while the expression of immune cell and pro-inflammatory cytokine-related gene sets was higher in the ankle joint, the TMJ showed increased expression of genes associated with energy consumption and bone resorption-related enzymes. These findings highlight the TMJ as a distinct anatomical site with heightened susceptibility to arthritis-related damage and emphasize the need for greater awareness and targeted research to improve disease management for affected individuals.

## Introduction

Inflammatory arthritis encompasses a group of autoimmune and inflammatory conditions that primarily affect joints, causing pain, swelling, and stiffness. These disorders differ in their presentation and underlying mechanisms but share common themes of immune-mediated joint inflammation and systemic involvement (1). Unlike osteoarthritis, which results from mechanical wear and tear, inflammatory arthritis is driven by immune dysfunction leading to progressive joint damage, deformity, and functional loss (2).

Rheumatoid arthritis (RA) is the most common inflammatory arthritis and a systemic autoimmune disorder targeting the synovial membrane of joints (3). Its pathogenesis involves the infiltration of autoreactive Th1 and Th17 cells, along with B cells, into the synovium. These immune cells release pro-inflammatory cytokines, such as tumor necrosis factor (TNF), interleukin-1 (IL-1), interleukin-6 (IL-6), and interleukin-17 (IL-17), as well as immune complexes, which amplify the inflammatory response (2, 4). This inflammatory milieu activates classical macrophages, neutrophils, and fibroblast-like synoviocytes (FLS) (5–7). Macrophages are key producers of pro-inflammatory cytokines, while neutrophils and FLS secrete matrix metalloproteinases (MMPs), leading to cartilage degradation (8). Additionally, Th cells and FLS express receptor activator of nuclear factor kappa-B ligand (RANKL), which, in combination with pro-inflammatory cytokines and immune complexes, drives the excessive formation and activation of bone-resorbing osteoclasts (9). These processes create a self-perpetuating cycle of inflammation, tissue destruction, and progressive joint damage. Antibodies against citrullinated proteins (ACPAs) are linked to a more severe and destructive course of disease (10). Additionally, evidence shows that bone loss in ACPA-positive individuals can begin prior to the onset of clinical symptoms, suggesting that these antibodies independently contribute to the initiation of skeletal damage (11, 12).

While inflammatory arthritis is commonly associated with the hands, wrists, and feet, it can also involve less-studied joints, such as the temporomandibular joint (TMJ), which plays a crucial role in jaw movement and functions like chewing and speaking. Typical symptoms of temporomandibular disorder (TMD) are pain, swelling and tenderness around the TMJ, limited mobility of the jaw, and noises during jaw movement (13). The prevalence of TMJ involvement in RA patients has been reported to range from 19% to 86% (14–16). These variations likely result from differences in diagnostic criteria, assessment methods, and RA disease duration (17). Moreover, clinical manifestations of TMJ arthritis are often silent and TMJ complaints may be overshadowed by RA symptoms somewhere else in the body (18). This makes TMJ arthritis challenging to diagnose and often requires physical examination in combination with imaging evaluations (19). Notably, increased prevalence of TMD pain has also been observed in individuals at risk of RA (17), highlighting its potential as an early indicator of disease rather than a feature of advanced disease stage.

Juvenile idiopathic arthritis (JIA), a chronic form of arthritis affecting children and adolescents from 6 months to 16 years (20), shows an especially high incidence of TMJ involvement, reaching up to 87% in some reports (21). In certain cases, the TMJ may be the only joint affected by JIA (22). However, the affected TMJ is often difficult to detect in early stages due to silent inflammation and osteochondral degradation (23, 24). Moreover, rheumatologists may have limited experience in diagnosing and managing TMJ-specific complications, in part due to a lack of specialized studies on the pathogenesis of inflammatory arthritis in the TMJ. Since the TMJ is a center of mandibular growth, affected children and adolescents develop dysgnathia and asymmetries, which often need orthodontic and sometimes surgical treatment. Therefore, understanding the pathomechanisms underlying TMJ involvement in inflammatory arthritis would help TMJ treatment and prevent TMJ destruction leading to severe craniofacial growth disturbances.

The TMJ is unique among joints in both its structure and function. Unlike other joints, which consist of primary cartilage, the mandibular condyle is composed of secondary cartilage (25). Moreover, the TMJ is covered by fibrocartilage rather than hyaline cartilage, providing greater durability and resistance to the multidirectional forces generated during jaw movement (25). Functionally, the TMJ is subjected to cyclic loading from activities like chewing and speaking, in contrast to the vertical weight-bearing forces typical of joints such as the knees or hips (26). These unique developmental and biomechanical characteristics may influence the manifestation and progression of arthritis in the TMJ. Consequently, studying the TMJ as a distinct entity is crucial for understanding its specific involvement in inflammatory arthritis.

Animal models are a well-established tool for investigating the pathological mechanisms of inflammatory arthritis, offering insights into cause-and-effect relationships that are difficult to obtain from human studies. However, the existing literature on mouse models demonstrating clear signs of TMJ inflammation, cartilage loss, and subchondral bone erosion remains scarce and partially contradictory (27). Thus, this study aimed to identify a suitable model for exploring the mechanisms underlying TMJ involvement in arthritis. The central question was whether the TMJ displays distinct pathological and molecular features compared to the well-studied ankle joint within an inflammatory arthritis context.

## Materials and methods

### Patient characteristics

To strengthen the clinical importance of our investigation, clinical data of patients with juvenile idiopathic arthritis (JIA)-related TMJ destruction were screened in the Department of Orthodontics Orofacial Orthopedics of the Universitätsklinikum Erlangen (UKER). This process identified a patient with symptoms of TMD such as pronounced pain and limited mobility of the mandibular jaw, who initially had consulted the clinic at the age of eight years. At the age of three, the patient had been diagnosed with JIA by a rheumatologist and treatment with pain killers and methotrexate was initiated. Later also anti-TNF medication Etanercept was prescribed. During acute phases of inflammatory arthritis and severe TMJ pain, the patient received intra-auricular injections of Triamcinolon-Hexacetonid alongside systematic treatment. Orthodontic treatment was initiated to alleviate TMJ pressure, enhance mandibular mobility and growth, and prevent further asymmetries and dentofacial disturbances. Therefore, extraoral and intraoral orthodontic diagnostics were performed as well as orthodontically indicated radiographic imaging, such as panoramic radiographs. Furthermore, magnetic resonance images (MRI) were used for diagnostics and for re-evaluation of the condylar resorption due to the inflammatory arthritis. Written informed consent for the use and publication of clinical images and data was obtained from both the patient and the parents, in accordance with the ethical guidelines of UKER and German Data Protection.

### MRI acquisition

Magnetic resonance imaging (MRI) was conducted using a Magnetom Area system (Siemens Healthineers, Erlangen, Germany) operating at a field strength of 1.5 Tesla. Imaging included sagittal proton density-weighted (PDw) sequences with the following parameters: repetition time (TR) = 2560 ms, echo time (TE) = 11 ms, flip angle (FA) = 150°, and a field of view (FOV) of 120 × 120 mm. The combination of these parameters optimized the signal-to-noise ratio and tissue contrast primarily driven by proton density differences, thereby enabling detailed assessment of the depicted anatomical structures. Additionally, coronal imaging comprised PDw, T1-weighted (T1w), and contrast-enhanced T1w sequences with fat suppression. Axial sequences included T2-weighted (T2w) and contrast-enhanced fat-suppressed T1w images. The combination of these sequences and orientations provided an adequate evaluation of both tissue morphology and contrast dynamics, improving diagnostic accuracy.

### Mice

The hTNFtg mice (strain Tg197 on a C57BL/6 background) have been previously described (28). Arthritis in hTNFtg mice was clinically assessed compared to littermate controls using grip strength, with a scoring system ranging from 4 (normal grip strength) to 0 (no grip on the cage bars), and by monitoring weight loss (29). Female mice were analyzed at 9–11 weeks of age. A group of 10 control mice was compared to 11 hTNFtg mice. All mice were housed in a temperature- and humidity-controlled environment with unrestricted access to food and water. The breeding of affected lines was conducted in accordance with the regulations of the FPZ animal facility (Franz-Penzoldt-Zentrum, Erlangen, Germany) and approved by the local ethics authorities.

### Micro-computed tomography imaging

Mouse skulls, hind paws, and mandibles were fixed in 4% PFA/PBS (pH 7.4) overnight and then transferred to 70% ethanol for preservation. All μCT imaging was performed using the cone-beam Desktop Micro Computer Tomograph μCT 40 (SCANCO Medical). The following settings were used for imaging calcified tissues in murine bones: kilovoltage (kVp) of 55, current of 145 μA, an integration time of 200 ms with 500 projections per 180° rotation, and an isotropic voxel size of 6.0 μm. Three-dimensional bone modeling was conducted using optimized grayscale thresholds within the Open VMS operating system (SCANCO Medical), with the extraction of bone parameters, including bone volume per total volume (BV/TV). The μCT image data were imported into Imaris software version 9.7.0 (Oxford Instruments) for surface rendering, using a smoothness parameter of 6 µm.

### Histological analysis

The hind paws from 5 littermate control mice and 5 hTNFtg mice, as well as the mandibles from 8 littermate controls and 6 hTNFtg mice, were fixed overnight in 4% PFA/PBS (pH 7.4). Tissues were then decalcified in 14% EDTA free acid with NH_4_OH (pH 7.2) for 14 days, until the bones became pliable. Serial paraffin sections (2 μm) were prepared and stained with hematoxylin and eosin stain (H&E; Carl Roth) and tartrate-resistant acid phosphatase (TRAP) stain using the Leukocyte Acid Phosphatase (TRAP) Kit (Merck; Cat# 387A). Inflammation area, bone erosion, and the number of osteoclasts were quantified using an Axio Lab.A1 microscope (Carl Zeiss) equipped with a digital camera and image analysis system (OsteoMeasure, Osteometrics).

### Flow cytometry

For cell isolation, ankle and temporomandibular joints from 5 littermate control mice and 5 hTNFtg mice were minced and incubated in RPMI medium containing 1 mg/ml collagenase A (Merck; Cat# 10103578001) and 0.1 mg/mL DNase I (Merck; Cat# 10104159001) at 37°C for 45 minutes with occasional mixing. After incubation, cells were washed, filtered through a 40 μm cell strainer, and incubated with TruStain FcX™ (anti-mouse CD16/32) antibody (clone 93; 1:200; BioLegend; Cat# 101320) for 10 minutes at 4°C. Following this, Zombie Red™ Fixable Viability dye (1:2000; BioLegend; Cat# 423110) was added, and cells were stained with an antibody cocktail for 20 minutes at 4°C. The antibodies used for membrane staining were: APC-eFluor™ 780-labelled anti-CD45 (cone 30-F11; 1:400; Thermo Fisher Scientific; Cat# 47-0451-82), FITC-labelled anti-CD11b (clone M1/70; 1:400; BD Pharmingen; Cat# 557396), BV421-labelled anti-CD19 (clone 1D3; 1:200; BD Pharmingen; Cat# 562701), PE-labelled anti-CD4 (clone GK1.5; 1:200; BD Pharmingen; Cat# 557308), APC-labelled anti-F4/80 (clone BM8; 1:200; BioLegend; Cat# 123116), and PerCP/Cyanine5.5-labelled anti-Ly-6G (clone 1A8; 1:400; BioLegend; Cat# 127616). After washing, cells were resuspended in FACS buffer (1x PBS with 2% FBS and 2 mM EDTA) for flow cytometric analysis. Flow cytometry was performed on a CytoFlex S flow cytometer (Beckman Coulter), and data were analyzed using Kaluza 2.1 software (Beckman Coulter).

### Bulk RNA-sequencing

RNA was extracted from the alveolar bone, TMJ, and ankle joint tissues of 4 hTNFtg mice and 4 littermate controls. Bulk RNA-seq was performed by Novogene (UK). According to Novogene’s protocol, 1 μg of RNA per sample was used as input material for RNA sample preparation. Sequencing libraries were generated using the NEBNext^®^ UltraTM RNA Library Prep Kit for Illumina^®^ (NEB, Cat# E7770) following the manufacturer’s instructions, with index codes added to assign sequences to individual samples. Sample clustering with index codes was performed as per the manufacturer’s guidelines. After cluster generation, the libraries were sequenced on an Illumina platform, generating paired-end reads. Clean reads were obtained by removing adapter sequences, poly-N reads, and low-quality reads from the raw data. All downstream analyses were based on these high-quality clean data. The filtered reads were aligned to the *Mus musculus* reference genome (GRCm39) using Kallisto (version 0.46.2). For each sample, paired-end reads were aligned to the indexed transcriptome with default settings and 4 threads per sample. The original RNA-seq data (fastq files) and the read counts have been deposited in the Gene Expression Omnibus (GEO) database with accession number GSE286871. Principal component analysis (PCA) and volcano plots were generated using the R packages tidyverse (version 2.0.0), plotly (version 4.10.4), and ggplot2 (version 3.5.1), respectively. Differential expression analysis between two conditions or groups (with four biological replicates per condition) was conducted using the limma (version 3.50.3) and edgeR (version 3.36.0) R packages. Gene expression data were normalized, and a linear model was fitted using the voom function to account for the mean-variance relationship. Differential expression analysis was conducted by applying empirical Bayes moderation using the eBayes function. A log2 fold change (logFC) cutoff of 1.0 was applied, and multiple testing correction was performed using the Benjamini–Hochberg (BH) method to adjust for false discovery rate (FDR). Genes with an adjusted *P*-value (*Padj*) less than 0.05 were considered differentially expressed. Heatmaps were created using the heatmap.2 function from the R package gplots (version 3.1.3.1), with a custom color palette. Significantly differentially expressed genes (*Padj* < 0.05, |log2FC| ≥ 1) were selected for clustering. Hierarchical clustering was performed using Pearson correlation for rows and Spearman correlation for columns. Gene expression values were scaled by row to emphasize relative differences. KEGG pathway analysis and visualization was performed using the pathview R package (version 1.42.0). Gene Set Enrichment Analysis (GSEA) based on Gene Ontology (GO) gene sets was conducted using the clusterProfiler package (version 4.10.1), utilizing log2 fold expression changes to identify pathways with consistent expression changes. Pathways were categorized based on normalized enrichment score (NES) and bubble plots were created with bubble size corresponding to the gene set size, and color reflecting the significance of the enrichment (adjusted *P*-value).

### RNA isolation and real-time PCR

RNA was extracted from alveolar bone, TMJ, and ankle joint tissue of 5 littermate controls and 5 hTNFtg mice using RNA-Solv^®^ Reagent (VWR; Cat# R6830-02) following the manufacturer’s protocol. Tissues were homogenized using the Precellys^®^ Steel Kit (VWR; Cat# P000910-LYSK0-A) on the Precellys^®^ 24 homogenizer (VWR). Genomic DNA contamination was removed from the extracted RNA using the DNase I Kit (Thermo Fisher Scientific; Cat# EN0521). The purified RNA was subsequently reverse transcribed into cDNA using the High-Capacity cDNA Reverse Transcription Kit (Thermo Fisher Scientific; Cat# 4368814). Quantitative real-time PCR was conducted with Takyon ROX SYBR 2X MasterMix dTTP Blue (Eurogentec; Cat# UF-RSMT-B0701) on the CFX96™ Real-Time System (Bio-Rad), employing primers listed in **Table 1**. Gene expression levels were normalized to *Actb*.

**Table 1:** Murine qPCR primers forward and reverse.

Gene	Forward	Reverse
*Actb*	5′TGTCCACCTTCCAGCAGATGT3′	5′AGCTCAGTAACAGTCCGCCTAGA3′
*Acp5*	5′CGACCATTGTTAGCCACATACG3′	5′TCGTCCTGAAGATACTGCAGGTT3′
*Csf1*	5′GCCTCCTGTTCTACAAGTGGAAG3′	5′ACTGGCAGTTCCACCTGTCTGT3′
*Il1b*	5′CAGGCAGGCAGTATCACTCA3′	5′AGGTGCTCATGTCCTCATCC3′
*Il6*	5′TACCACTTCACAAGTCGGAGGC3′	5′CTGCAAGTGCATCATCGTTGTTC3′
*Il17*	5′TAACTCCCTTGGCGCAAAAG3′	5′TCTTCATTGCGGTGGAGAGTC3′
*Mmp9*	5′GCTGACTACGATAAGGACGGCA3′	5′TAGTGGTGCAGGCAGAGTAGGA3′
*Nfatc1*	5′GGTGCCTTTTGCGAGCAGTATC3′	5′CGTATGGACCAGAATGTGACGG3′
*Tnfa*	5′CGGCATGGATCTCAAAGACAAC3′	5′AGATAGCAAATCGGCTGACG3′
*Tnfsf11*	5′GTGAAGACACACTACCTGACTCC3′	5′GCCACATCCAACCATGAGCCTT3′

### Statistics

All statistical analyses were conducted using GraphPad Prism Software 9. Data are presented as the mean ± standard error of the mean (SEM). Statistical significance was determined using an unpaired two-tailed t-test. Detailed statistical information (e.g., number of animals per group) is provided in the figure legends. *P*-values less than 0.05 were considered statistically significant.

## Results

### The hTNFtg arthritis mouse model shows TMJ involvement similar to that seen in arthritic patients

To investigate the pathophysiology of TMJ involvement in inflammatory arthritis, we utilized mouse models, as human studies are limited to describe disease mechanisms. We characterized TMJ involvement in three inflammatory arthritis models: collagen-induced arthritis (CIA), K/BxN serum-transfer arthritis (STA), and human TNF-α transgenic mice (hTNFtg). The CIA model reflects the autoimmune nature of RA, characterized by T and B cell activation and the production of autoantibodies (30). The STA model represents acute inflammation primarily driven by the innate immune response and pathogenic autoantibodies (31). In contrast, the hTNFtg model simulates chronic TNF-mediated inflammation, which is solely cytokine-driven and does not involve autoantibodies (28).

In the CIA model, arthritis was induced by immunizing susceptible DBA/1 mice with type II collagen (CII) emulsified in an adjuvant, leading to an autoimmune response and the production of autoantibodies against collagen (30). As shown in **Supplementary Figure 1A**, this resulted in joint pathology, evidenced by increased arthritis scores, which included swelling severity of the toes, paw, and ankle. Despite successfully induced arthritis, CIA mice showed no TMJ alterations. This finding was confirmed by microcomputed tomography (µCT), which revealed no changes in bone volume per total volume (BV/TV) in the mandibular condyle (**Supplementary Figures 1B, C**). Additionally, histomorphometric analysis of immune cell infiltration and cartilage degradation using H&E staining, as well as osteoclast-mediated subchondral bone erosion assessed by TRAP staining, showed no differences in the TMJ between healthy DBA/1 mice and CIA mice (**Supplementary Figure 1D**).

The STA model relies on the injection of serum collected from arthritic K/BxN mice, which contains anti-glucose-6-phosphate isomerase immune complexes that induce transient arthritis in recipient mice by activating the innate immune system (31). Although STA mice developed arthritis (**Supplementary Figure 2A**), the TMJ remained unaffected. This was confirmed through µCT and histological analyses, which showed no changes in bone structure, immune cell infiltration, or cartilage integrity (**Supplementary Figures 2B–D**).

**Figure 1:**
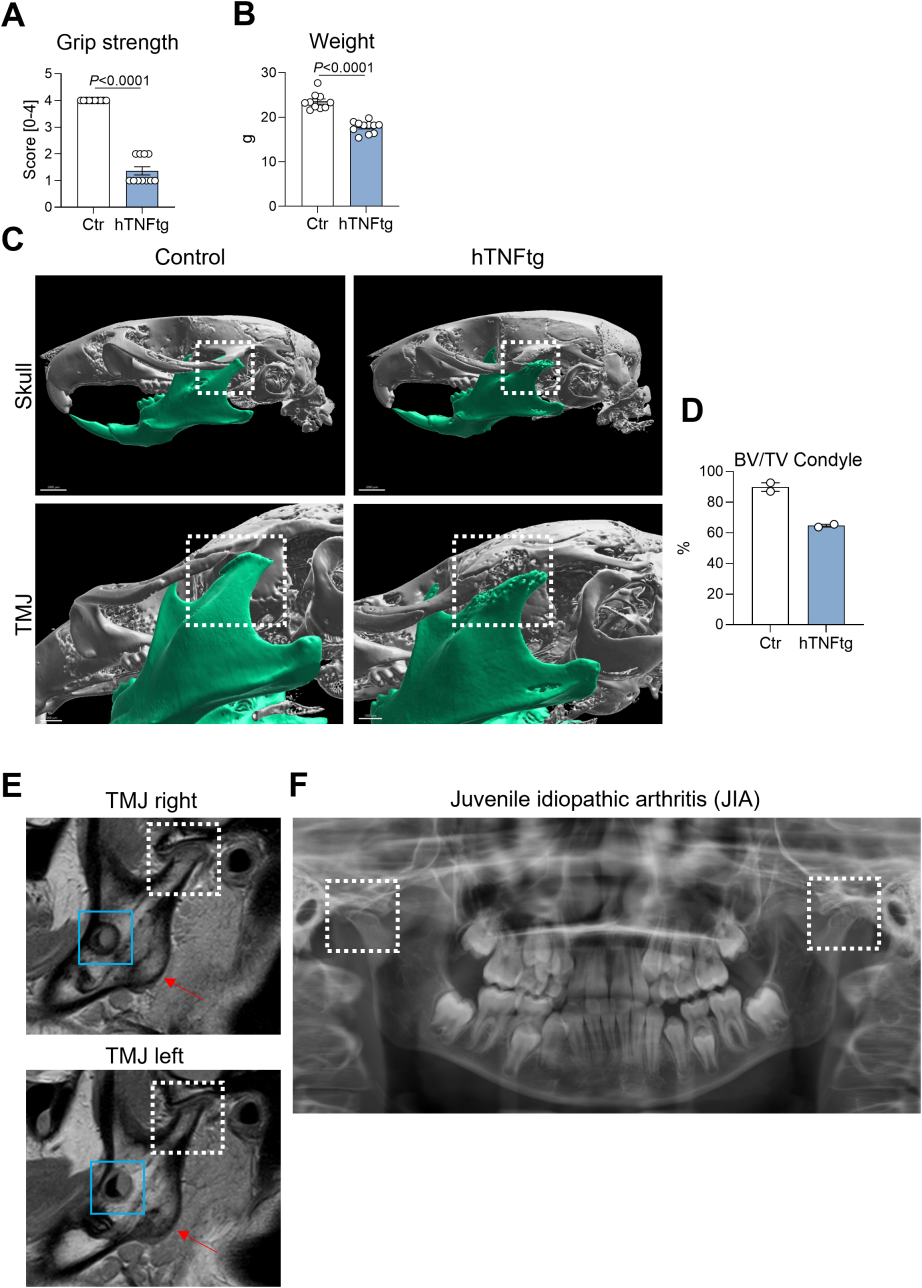
The hTNFtg arthritis mouse model demonstrates TMJ involvement with characteristics resembling those observed in arthritic patients. **(A)** Arthritis severity in hTNFtg mice was assessed in comparison to littermate controls using grip strength measurements (n=10-11). **(B)** Arthritis severity in hTNFtg mice was evaluated relative to littermate controls based on weight loss (n=10-11). **(C)** Micro-computed tomography (μCT) images of the skull illustrate comparisons between hTNFtg mice and littermate controls. The scale bar represents 2 mm in the upper images and 1 mm in the lower images. **(D)** Bone volume per total volume (BV/TV) of the mandibular condyle was analyzed in hTNFtg mice versus littermate controls by μCT (n=2). **(E)** Representative magnetic resonance images (MRI) of a 14-year-old patient diagnosed with juvenile idiopathic arthritis (JIA). Anatomical landmarks are highlighted for orientation: white dashed rectangle—condylar flattening with pronounced bone erosion and cartilage loss due to inflammation and osteochondylar resorption of the right and left temporomandibular joint (TMJ); blue rectangle—developing third molars (teeth 38 and 48); red arrow—mandibular angle. **(F)** Orthodontically indicated panoramic radiograph from the same patient two years earlier. Bilateral condylar resorption is already evident (white dashed rectangle) explaining clinical symptoms of severe TMJ pain, loss of mandibular mobility and dentofacial growth disturbances. Data are shown as mean ± SEM. Symbols represent individual mice. *P* values were determined by unpaired two-tailed *t* test for single comparisons. *P*-values less than 0.05 were considered statistically significant.

In the hTNFtg mouse model, the overexpression of human TNF-α, a key pro-inflammatory cytokine implicated in the pathogenesis of RA (32), leads to typical symptoms of inflammatory polyarthritis (28). Disease severity was demonstrated by reduced grip strength and weight loss (**Figures 1A, B**). Consistent with the literature (33), severe bone erosion in the TMJ was observed, as evidenced by µCT imaging of the mouse skull (**Figure 1C**) and a reduction in BV/TV in the mandibular condyle (**Figure 1D**). Importantly, the TMJ phenotype observed in hTNFtg mice closely resembled the TMJ involvement seen in a juvenile idiopathic arthritis (JIA) patient (**Figures 1E, F**). MRI revealed a severe inflammatory arthritis, which already caused excessive bone erosion and cartilage loss in both TMJs, explaining the pain and loss of mandibular mobility (**Figure 1E**). Orthodontically indicated X-rays supported these findings, demonstrating mandibular condyle resorption of both joints (**Figure 1F**). These data show that hTNFtg mice serve as a suitable model for studying inflammatory arthritis-induced TMD.

### The TMJ of hTNFtg mice exhibits inflammation, cartilage degradation, and bone erosion

**Figure 2:**
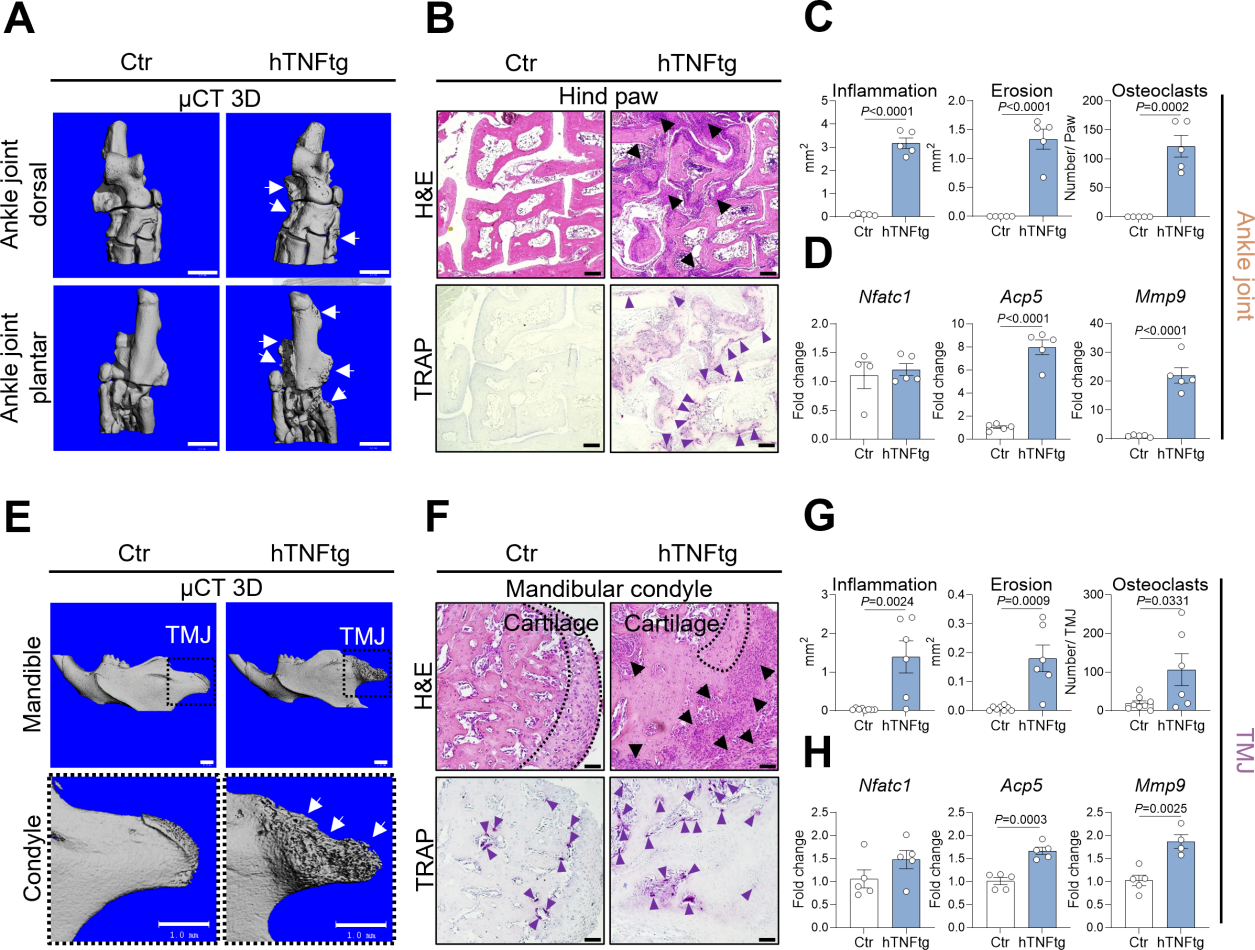
The TMJ of hTNFtg mice exhibits inflammation, cartilage degradation, and bone erosion. **(A)** Representative μCT images showing bone erosion (white arrows) in the hind paws of hTNFtg mice compared to littermate controls. The scale bar indicates 1 mm. **(B)** Hematoxylin and eosin (H&E) and tartrate-resistant acid phosphatase (TRAP) staining of the hind paws in hTNFtg mice versus littermate controls. Inflammation is represented by black triangles, while bone-resident osteoclasts are depicted with purple triangles. The scale bar represents 50 μm. **(C)** Histomorphometric analysis quantifying inflammation, bone erosion, and osteoclast numbers in the hind paws of hTNFtg mice compared to littermate controls (n=5). **(D)** mRNA expression levels of *Nfatc1*, *Acp5*, and *Mmp9* in the ankle joints of hTNFtg mice relative to littermate controls (n=4-5). **(E)** Representative μCT images illustrating bone erosion (white arrows) in the mandibular condyle of hTNFtg mice compared to littermate controls. The scale bar represents 1 mm. **(F)** H&E and TRAP staining of the mandibular condyle in hTNFtg mice and littermate controls. Black triangles indicate inflammation, whereas purple triangles represent bone-resident osteoclasts. The scale bar represents 50 μm. **(G)** Histomorphometric quantification of inflammation, bone erosion, and osteoclast numbers in the mandibular condyle of hTNFtg mice compared to littermate controls (n=6–8). **(H)** mRNA expression of *Nfatc1*, *Acp5*, and *Mmp9* in the temporomandibular joint (TMJ) of hTNFtg mice relative to littermate controls (n=4-5). Data are shown as mean ± SEM. Symbols represent individual mice. *P* values were determined by unpaired two-tailed *t* test for single comparisons. *P*-values less than 0.05 were considered statistically significant.

Next, we aimed to determine the cellular and molecular mechanisms driving the severe bone erosion observed in the TMJ of hTNFtg animals, comparing TMJ manifestations to the well-characterized phenotype of the ankle joint (**Figures 2A, E**). Similar to the ankle joint, the TMJ of hTNFtg mice exhibited extensive immune cell infiltration, as demonstrated by H&E staining (**Figures 2B, C, F, G)**. Moreover, H&E staining demonstrated severe cartilage degradation in the TMJ of hTNFtg mice (**Figure 2F**). The pronounced subchondral bone erosion in the arthritic ankle joint and TMJ was associated with an increased number of mature bone-resident osteoclasts, quantified by TRAP staining (**Figures 2B, C, F, G**). Furthermore, mRNA analysis revealed significantly elevated expression of bone-resorptive enzymes such as *Acp5* (encoding TRAP) and *Mmp9* in the ankle joint as well as the TMJ of hTNFtg mice (**Figures 2D, H**). Interestingly, unlike the hind paws, which lacked TRAP-positive cells in healthy conditions, the TMJ exhibited osteoclast-mediated bone remodeling even under steady-state conditions (**Figures 2B, C, F, G**). This may explain why the expression of osteoclast-associated genes between healthy and arthritic condition was eight- to twentyfold higher in the ankle joint, compared to the more modest twofold increase in the TMJ (**Figures 2D, H**). Despite this disparity, subchondral bone degradation in hTNFtg mice was even more pronounced in the TMJ than in the ankle joint (**Figures 2A, E**).

### The TMJ of hTNFtg mice displays a unique pattern of immune cell infiltration and pro-inflammatory cytokine expression

**Figure 3:**
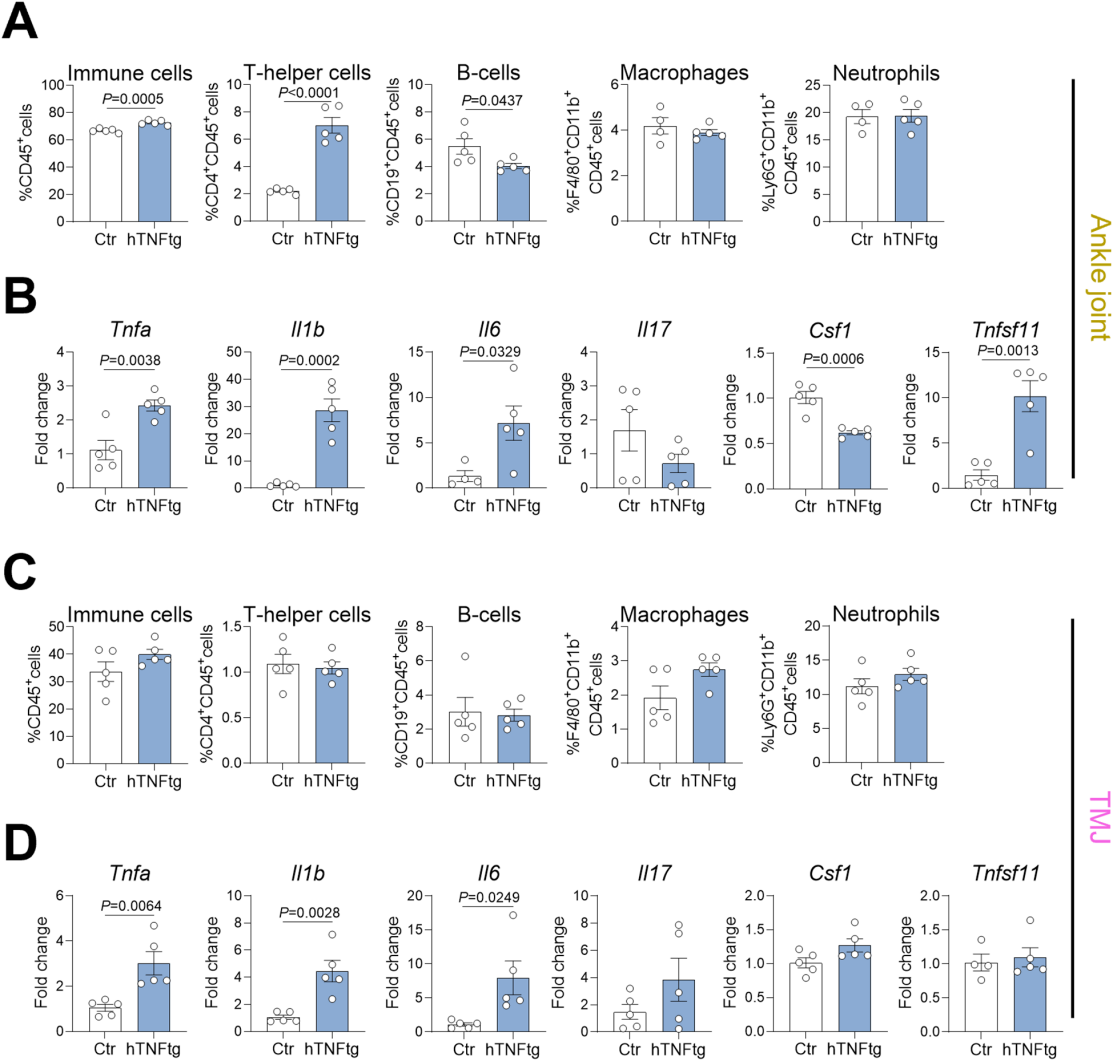
The TMJ of hTNFtg mice displays a unique pattern of immune cell infiltration and pro-inflammatory cytokine expression. **(A)** Proportions of CD45^+^ immune cells, CD4^+^ T helper cells, CD19^+^ B cells, CD11b^+^F4/80^+^ macrophages, and CD11b^+^Ly6G^+^ neutrophils in the ankle joints of hTNFtg mice compared to littermate controls (n=4-5). **(B)** mRNA expression levels of *Tnfa*, *Il1b*, *Il6*, *Il17*, *Csf1*, and *Tnfsf11* in the ankle joints of hTNFtg mice relative to littermate controls (n=4-5). **(C)** Proportions of CD45^+^ immune cells, CD4^+^ T helper cells, CD19^+^ B cells, CD11b^+^F4/80^+^ macrophages, and CD11b^+^Ly6G^+^ neutrophils in the TMJ of hTNFtg mice compared to littermate controls (n=5). **(D)** mRNA expression levels of *Tnfa*, *Il1b*, *Il6*, *Il17*, *Csf1*, and *Tnfsf11* in the TMJ of hTNFtg mice relative to littermate controls (n=4-5). Data are shown as mean ± SEM. Symbols represent individual mice. *P* values were determined by unpaired two-tailed *t* test for single comparisons. *P*-values less than 0.05 were considered statistically significant.

To further investigate the pathomechanism of arthritis-mediated TMD, we performed flow cytometry on cells isolated from the TMJ and compared them to those from the ankle joint in control and hTNFtg mice (**Supplementary Figure 3**). Notably, the ankle joint exhibited a markedly increased proportion of CD4+ Th cells in the context of arthritis, while the percentage of CD19+ B cells was reduced, and the number of macrophages and neutrophils was unaffected (**Figure 3A**). The TMJ maintained a normal distribution of CD4+ Th cells, CD19+ B cells, and neutrophils with a tendential increase in macrophages (**Figure 3C**). Regarding the expression of pro-inflammatory cytokines, both the ankle joint and the TMJ demonstrated significantly elevated levels of *Tnfa*, *Il1b*, and *Il6* (**Figures 3B, D**). Interestingly, only the ankle joint showed increased expression of the pro-osteoclastogenic cytokine RANKL (*Tnfsf11* gene), while M-CSF expression (*Csf1* gene) was even downregulated (**Figure 3B**). In contrast, the TMJ displayed no changes in RANKL or M-CSF expression, but showed a tendential increase in *Il17* expression (**Figure 3D**). These findings suggest that the overall inflammatory response is more pronounced in the ankle joint compared to the TMJ in hTNFtg mice.

### Bulk RNA sequencing reveals significant changes in the expressional profile of the TMJ as compared to the ankle joint of hTNFtg mice

**Figure 4:**
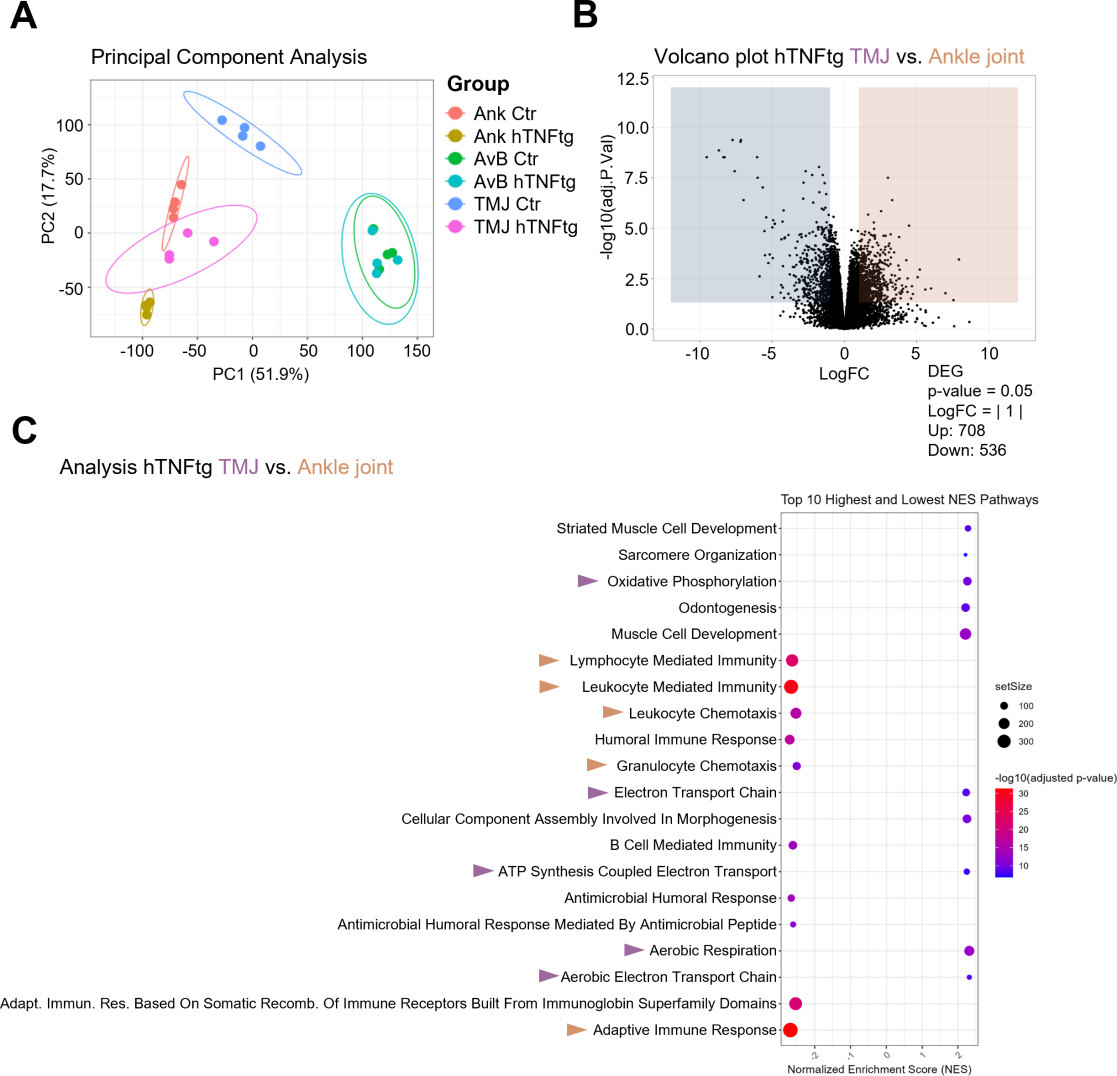
Bulk RNA sequencing reveals significant changes in the expressional profile of the TMJ as compared to the ankle joint of hTNFtg mice. **(A)** Principal component analysis (PCA) illustrating the distribution of individual samples across six groups (n=4): Ankle Control (Ank Ctr), Ankle hTNFtg (Ank hTNFtg), Alveolar Bone Control (AvB Ctr), Alveolar Bone hTNFtg (AvB hTNFtg), TMJ Control (TMJ Ctr), and TMJ hTNFtg. **(B)** Volcano plot depicting differentially expressed genes (DEGs) between Ankle hTNFtg and TMJ hTNFtg groups, with downregulated DEGs shown in blue and upregulated DEGs in red. **(C)** Gene Ontology (GO) enrichment bubble plot comparing Ankle hTNFtg and TMJ hTNFtg groups. The most relevant enriched pathways are highlighted with pink arrows when upregulated in the hTNFtg TMJ group and with yellow arrows when upregulated in the hTNFtg ankle group. Differential expression analysis was conducted with a log2 fold change threshold of 1.0. *P*-values were adjusted using the Benjamini–Hochberg method to control the false discovery rate (FDR). Genes with an adjusted *P*-value (*Padj*) less than 0.05 were considered differentially expressed.

To distinguish the arthritis-mediated immune response in the TMJ from the well-characterized ankle joint, we performed bulk RNA sequencing. In addition to TMJ and ankle joint tissue, we collected alveolar bone samples from control and hTNFtg mice, as previous studies have shown that inflammatory arthritis contributes to alveolar bone loss (34). While the ankle joint and TMJ displayed significant changes in gene expression under arthritic conditions compared to controls, the gene expression in the alveolar bone remained unaffected by chronic TNF-mediated arthritis, as revealed by principal component analysis (**Figure 4A**).

Volcano plots and Gene Ontology (GO)-enrichment analyses confirmed that both the ankle joint and TMJ exhibited an enhanced inflammatory response in the hTNFtg mice as compared to littermate controls. Heatmaps of differentially expressed genes (DEGs) and KEGG pathways comparing healthy and arthritic ankle joints and TMJs corroborated the histomorphometry and qPCR data, showing increased expression of pro-inflammatory genes as well as pro-osteoclastogenic genes in both joints under inflammatory conditions (**Supplementary Figures 4**-**9**).

Interestingly, the gene expression profile of the arthritic TMJ was markedly distinct from that of the arthritic ankle joint, as shown by volcano plot, depicting 708 upregulated and 536 downregulated genes (**Figure 4B**). GO-enrichment analysis further revealed that pathways associated with innate and adaptive immune response were downregulated in the arthritic TMJ compared to the arthritic ankle joint. In contrast, pathways linked to energy metabolism and jaw tissue-specific pathways were upregulated (**Figure 4C**).

**Figure 5:**
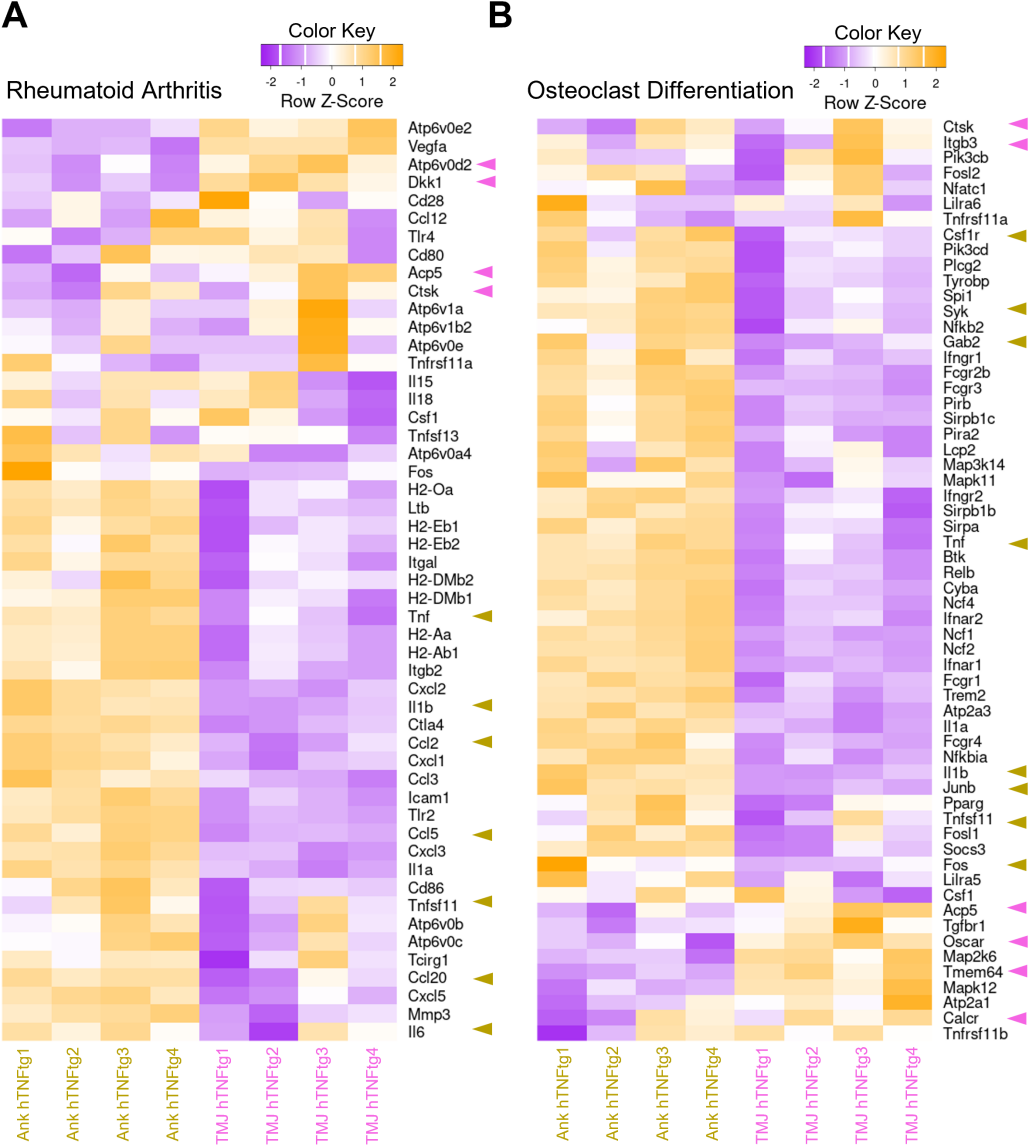
Differential expression of pro-inflammatory and pro-osteoclastogenic genes in the TMJ compared to the ankle joint of hTNFtg mice. **(A)** Heatmap displaying rheumatoid arthritis-associated differentially expressed genes (DEGs) between the Ankle hTNFtg and TMJ hTNFtg groups. **(B)** Heatmap presenting osteoclast differentiation-associated DEGs between the Ankle hTNFtg and TMJ hTNFtg groups. The most relevant genes are highlighted with pink arrows when upregulated in the hTNFtg TMJ group and with yellow arrows when upregulated in the hTNFtg ankle group. Differential expression analysis was performed using a log2 fold change cutoff of 1.0. *P*-values were adjusted using the Benjamini–Hochberg method to control the false discovery rate (FDR). Genes with an adjusted *P*-value (*Padj*) less than 0.05 were classified as differentially expressed.

Overall, the arthritic ankle joint exhibited elevated inflammation levels, including increased expression of the chemokines CCL2, CCL5, and CCL20, which are crucial for T cell recruitment (**Figure 5A**). This may explain the accumulation of CD4+ Th cells in the arthritic ankle joint. Notably, gene sets associated with osteoclast-mediated bone resorption, such as *Oscar*, *Ctsk*, *Acp5*, and *Atp6v0d2*, were higher expressed in the arthritic TMJ (**Figures 5A, B**). This may account for the pronounced subchondral bone erosion observed in the TMJ of hTNFtg mice. Furthermore, the gene encoding the Wnt signaling inhibitor Dickkopf-1 (Dkk1) was specifically upregulated in the arthritic TMJ compared to the arthritic ankle joint (**Figure 5A**), suggesting additional inhibition of osteoblast-mediated bone formation upon inflammatory arthritis in the TMJ.

**Figure 6:**
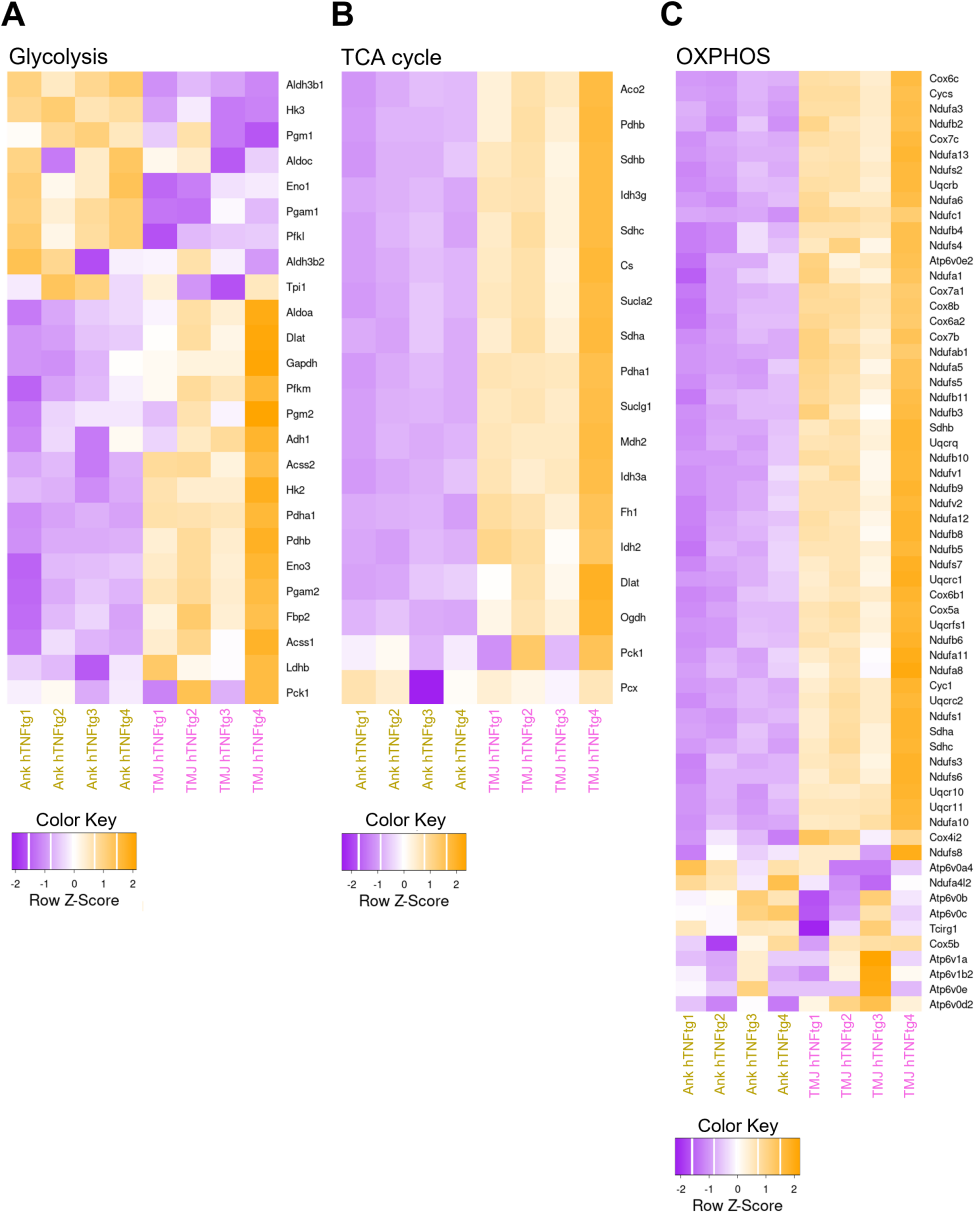
Elevated expression of metabolic genes in the TMJ relative to the ankle joint in hTNFtg mice. **(A)** Heatmap illustrating glycolysis-related differentially expressed genes (DEGs) between the Ankle hTNFtg and TMJ hTNFtg groups. **(B)** Heatmap showing TCA cycle-related DEGs between the Ankle hTNFtg and TMJ hTNFtg groups. **(C)** Heatmap depicting oxidative phosphorylation (OXPHOS)-related DEGs between the Ankle hTNFtg and TMJ hTNFtg groups. Differential expression analysis was conducted with a log2 fold change threshold of 1.0. *P*-values were adjusted using the Benjamini–Hochberg method to control the false discovery rate (FDR). Genes with an adjusted *P*-value (*Padj*) less than 0.05 were considered differentially expressed.

Additionally, genes involved in energy metabolism, including glycolysis, the TCA cycle, and oxidative phosphorylation (OXPHOS), were significantly upregulated in the TMJ (**Figures 6A–C**). This elevated energy demand likely reflects the activation of the immune system and the heightened activity of osteoclasts.

**Figure 7:**
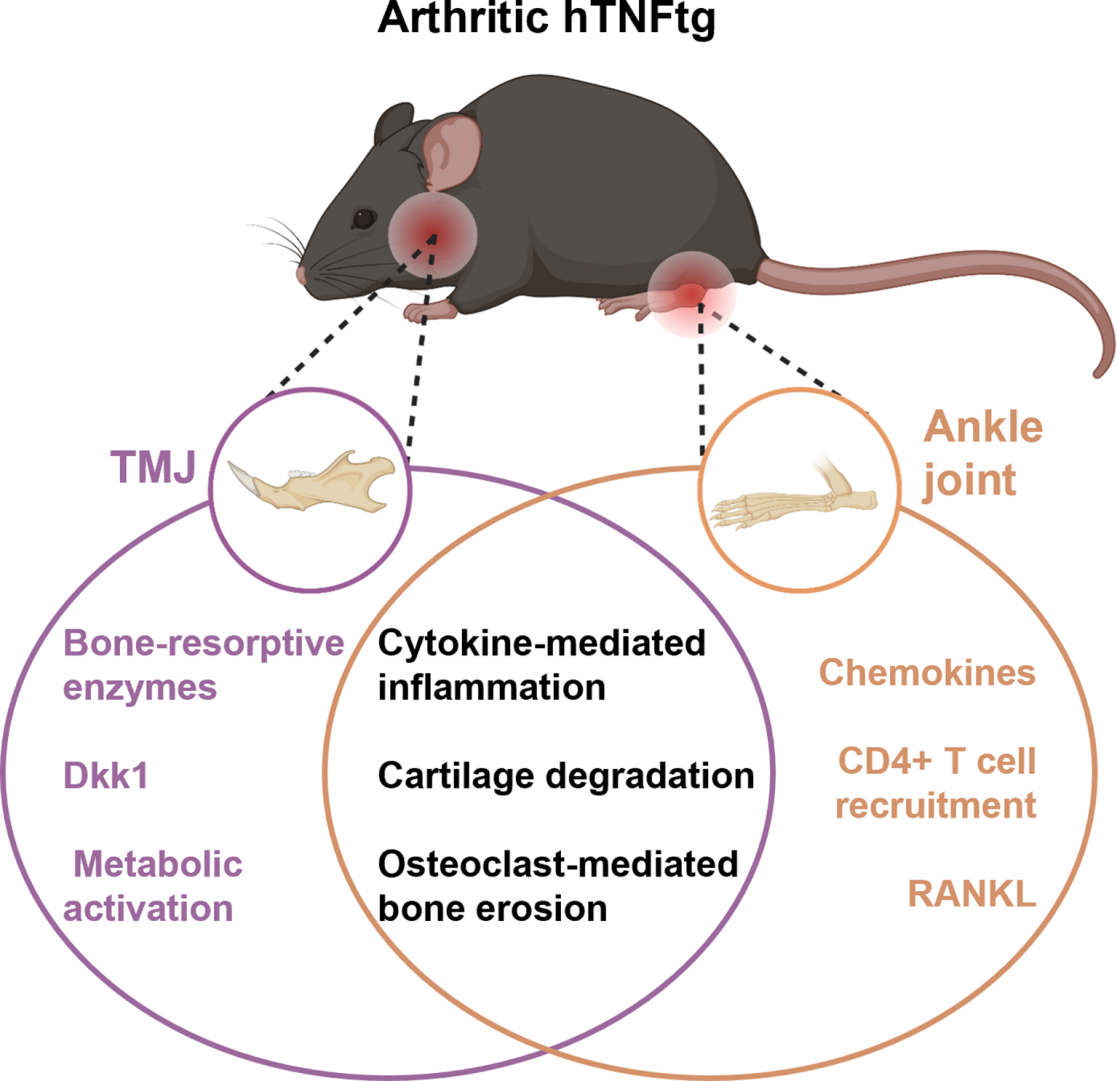
Graphical overview of distinct and shared molecular pathways contributing to joint damage in different anatomical sites in the hTNFtg model. While both joints show inflammation and bone/cartilage damage, the TMJ is characterized by metabolic and enzyme-driven bone resorption, whereas the ankle exhibits stronger immune cell recruitment and chemokine signaling. Figure partially generated with BioRender.

In summary, these findings demonstrate that TNF-mediated inflammatory arthritis affects the TMJ, leading to immune cell infiltration, cartilage degradation, and severe subchondral bone erosion. Mechanistically, TMJ involvement appears to elicit a unique inflammatory response distinct from the pathomechanism observed in the ankle joint. While both joints exhibit inflammation and bone tissue degradation, the TMJ is more severely impacted. The ankle joint shows higher levels of pro-inflammatory cytokines and Th cell infiltration, whereas the TMJ displays enhanced osteoclast activity and metabolic activation (**Figure 7**).

## Discussion

TMJ involvement in inflammatory arthritis, particularly JIA, is a significant yet underexplored complication. The pronounced TMJ erosion observed in JIA patients underscores the unique susceptibility of this joint to inflammatory damage, often resulting in pain, functional impairment, and long-term deformities (35). The distinct anatomical and functional properties of the TMJ, including its role in mastication and its exposure to mechanical loading, likely contribute to its vulnerability. There are some structural differences in the TMJ between mice and humans. Differences are evident in both the thickness of the articular disc and in biomechanical properties. In humans, the stiffness of the disc and the mandibular condylar cartilage is more comparable, whereas in mice, the articular disc exhibits significantly lower stiffness than the cartilage. This reduced stiffness in the murine disc leads to greater congruency during jaw movement and contributes to joint protection by reducing mechanical overload (36). Despite these anatomical distinctions, this study highlights the translational relevance of using mouse models, especially the hTNFtg model, to study TMJ pathophysiology.

Various models of osteoarthritis affecting the TMJ have been described (37). However, despite the numerous murine polyarthritis models available, only a few studies have addressed TMJ involvement. One study utilizing the K/BxN mouse model reported TMJ alterations, although the observed phenotype was relatively mild (38). In contrast, a study by Safi et al. did not identify any histological or immunohistochemical evidence of inflammation in the TMJ of K/BxN serum transfer arthritis mice (26). Bone erosion and cartilage loss in the TMJ have been observed in previous studies using hTNFtg mice (27, 33). However, these studies lacked an in-depth investigation into the cellular and molecular mechanisms driving TMJ inflammation, cartilage degradation, and subchondral bone erosion.

Our study confirms a notable difference in TMJ involvement among various inflammatory arthritis models. Specifically, the hTNFtg model exhibited pronounced TMJ pathology, whereas the K/BxN serum-transfer arthritis and collagen-induced arthritis models did not show similar TMJ alterations, despite causing cartilage and bone damage in the paws. In the hTNFtg model, the mouse TNF-α gene is replaced with the human TNF-α gene, with expression driven by the mouse endogenous promoter. This results in arthritis features closely resembling those of human RA, which may explain why TMJ arthritis is observed exclusively in this model. The presence of autoantibodies does not appear to be the primary driver of TMJ arthritis in these mouse models, as the hTNFtg model, unlike CIA and STA, is solely cytokine-driven. Additionally, the detected divergence may be attributed to the central role of TNF-α in mediating TMJ-specific pathology. Unlike the STA and CIA models, where the inflammatory response is transient or primarily driven by other immune pathways (39), the chronic overexpression of TNF-α in hTNFtg mice likely drives persistent inflammation, cartilage degradation, and osteoclast activation in the TMJ. The well-documented role of TNF-α in promoting osteoclastogenesis (40) and inhibiting osteoblast function (41) may be particularly impactful in the TMJ, where baseline bone remodeling activity is inherently higher than in other joints (42). The discovery of the pivotal role TNF-α plays in RA pathogenesis has revolutionized the treatment of the disease. Anti-TNF therapies have become a cornerstone in the management of moderate-to-severe RA, particularly in patients who do not respond adequately to conventional disease-modifying antirheumatic drugs like methotrexate (43). In the future, exploring the responsiveness of RA patients with TMJ involvement to targeted anti-TNF therapy would be highly relevant.

The TMJ in hTNFtg mice exhibited severe subchondral bone erosion, which was even more pronounced than in the ankle joint. This can be associated with the overactivation of osteoclasts alongside the inhibition of osteoblasts. The upregulation of genes such as *Acp5* (encoding TRAP), *Ctsk*, and *Atp6v0d2* indicate enhanced osteoclast activity in the arthritic TMJ. In contrast, RANKL (*Tnfsf11*) and M-CSF (*Csf1*), key drivers of osteoclastogenesis, were not elevated in the arthritic TMJ, suggesting alternative mechanisms of osteoclast induction. Pro-inflammatory cytokines, such as TNF-α and IL-1, can directly stimulate osteoclastogenesis by binding to their respective receptors, TNFR1 and IL-1R, on osteoclasts (9). Moreover, osteoclasts can be promoted by TREM2- or OSCAR-mediated costimulatory signaling pathways through the induction of DAP12/FcRγ-Syk-PLCγ signaling cascades that activate calcium signaling and NFATc1 (44, 45). Indeed, *Oscar* expression was upregulated in the TMJ compared to the ankle joint of hTNFtg mice. Interestingly, in addition to osteoclast activation, the arthritic TMJ may also exhibit osteoblast inhibition, as evidenced by elevated expression of Dickkopf-1 (DKK1). DKK1, a Wnt signaling inhibitor, is known to promote osteoclastogenesis and suppress osteoblast differentiation (46). In summary, the increased subchondral bone erosion observed in the arthritic TMJ is likely driven by an imbalance between osteoclast-mediated bone resorption and osteoblast-driven bone formation at the site of erosion.

Despite the significant inflammation, cartilage degradation, and subchondral bone loss observed in the arthritic TMJ, the overall expression of inflammation-related mediators, including TNF-α, IL-1β, and IL-6, was higher in the arthritic ankle joint than in the TMJ. Additionally, Th cell accumulation was detected exclusively in the arthritic ankle joint. This discrepancy can be attributed to the pronounced upregulation of lymphocyte-recruiting chemokines, such as CCL2, CCL5, and CCL20, in the ankle joint compared to the TMJ of hTNFtg mice (47). The underlying reason for this joint-specific manifestation remains unclear.

Although not directly analyzed, fibroblasts may play a pivotal role in the differing responses of the ankle joint and TMJ to inflammatory arthritis. As key stromal cells in the synovium, fibroblasts are capable of producing inflammatory mediators, matrix-degrading enzymes, and factors such as RANKL and DKK1 that influence both osteoclast and osteoblast activity (2). Differential gene expression in synovial fibroblasts from the TMJ and knee joints has been observed following exposure to mechanical stress (48). Similarly, TMJ fibroblasts might exhibit a unique phenotype that increases the joint’s susceptibility to bone erosion in response to inflammatory arthritis. Thus, future research should focus on identifying fibroblast-specific markers and elucidating their functional roles in inflammatory arthritis-driven TMD.

RNA sequencing revealed significant upregulation of metabolic pathways in the arthritic TMJ, including glycolysis, the TCA cycle, and OXPHOS. This heightened metabolic activity likely reflects the energy demands of activated immune cells, osteoclasts, and synovial fibroblasts in the inflamed TMJ. Enhanced metabolic activity may drive the severe bone erosion observed, as osteoclast function is closely tied to energy availability (49–51).

While this study provides valuable insights into the molecular and structural changes associated with arthritis-induced TMD, a few limitations should be considered. Although the sample size was appropriate for exploratory analysis, future validation in larger cohorts would help to support the broader applicability of the findings. Additionally, while the study focused on transcriptomic data, complementary protein-level conformation of key gene expression changes such as CCL2/5, DKK1, RANKL, OSCAR, and TRAP could further enhance the mechanistic interpretation.

Nevertheless, the study offers valuable insights into the specific mechanisms driving inflammatory arthritis in the TMJ, with a focus on TNF-α-mediated inflammation, osteoclast activity, and metabolic dysregulation. Our data support the notion that the TMJ is a distinct anatomical site with unique susceptibility to arthritis-induced damage, highlighting the need for joint-specific research and therapeutic strategies. Given its particular inflammatory and metabolic profile, treatments effective in other joints may not directly translate to the TMJ. Accordingly, targeting TNF-α, DKK1, or specific metabolic pathways may offer promising therapeutic avenues for patients with inflammatory arthritis-associated TMD. Future research should investigate the role of fibroblasts, explore metabolic intervention strategies, and evaluate treatment efficacy within this specific disease context.

## Data Availability

The datasets presented in this study can be found in online repositories. The names of the repository/repositories and accession number(s) can be found below: https://www.ncbi.nlm.nih.gov/geo/query/acc.cgi?acc=GSE286871.
