# Supplementary material for: A multidimensional analysis of temporomandibular joint and ankle joint erosion in inflammatory arthritis
Source: Front Immunol. 2025 Jul 18;16:1560723. doi: 10.3389/fimmu.2025.1560723 (PMC12313558; doi:10.3389/fimmu.2025.1560723)
Supplement: Supplementary file 1 [file DataSheet1.pdf]

Supplementary Material

A multidimensional analysis of temporomandibular joint and ankle joint erosion in inflammatory arthritis

Darja Andreev[1,2,3]*, Pauline Porschitz[1], Daniela Weidner[2,3,4], Rui Song[2,3], Matthias Weider[5], Georg Schett[2,3], Lina Gölz[5], Aline Bozec[2,3]

[1]Center for Regenerative Therapies Dresden (CRTD), Technische Universität (TU) Dresden, 01307 Dresden, Germany.

[2]Department of Medicine 3 - Rheumatology and Immunology, Friedrich-Alexander-Universität Erlangen-Nürnberg (FAU) and Universitätsklinikum Erlangen (UKER), 91054 Erlangen, Germany.

[3]Deutsches Zentrum Immuntherapie (DZI), FAU and UKER, 91054 Erlangen, Germany.

[4]Exploratory Research Unit, Optical Imaging Centre Erlangen, FAU, 91058 Erlangen, Germany.

[5]Dental Clinic 3 – Department of Orthodontics and Orofacial Orthopedics, FAU and UKER, 91054 Erlangen, Germany.

*** Correspondence:**
Dr. Darja Andreev
Darja.andreev@tu-dresden.de

1 Supplementary Data

1.1 Supplementary Materials and Methods

Collagen-induced arthritis

DBA/1 males (7 weeks old) purchased from Janvier Labs were immunized via subcutaneous injection at the base of the tail with 100 µL of 0.25 mg chicken type II collagen (CII; Merck, Cat# C9301) in complete Freund's adjuvant (CFA; Merck, Cat# F5881), containing 5 mg/mL killed *Mycobacterium tuberculosis* (H37Ra). Twenty-one days after the primary immunization, the mice received a secondary boost with the same amount of CII emulsified in CFA, administered subcutaneously at the base of the tail near the initial injection site. Clinical scores for each paw were assessed every third day and rated on a scale of 0–4 as described [1]. CIA mice were analyzed on day 42 post-immunization at an age of 13 weeks. Six mice treated with CIA were compared to six control mice that received mock treatment.

K/BxN serum-transfer arthritis

Wild-type BALB/cJRj females (11 weeks old) were purchased from Janvier Labs and injected intraperitoneally with 200 µl pooled serum from arthritic adult K/BxN mice as described [2]. Development of arthritis was evaluated for each paw using a semi-quantitative scoring system (0–4 per

paw; maximum score of 16) as previously described [3]. Mice were analyzed at day 12 post-serum transfer at an age of 13 weeks. Five mice treated with STA were compared to five control mice that received PBS treatment.

2 Supplementary Figures

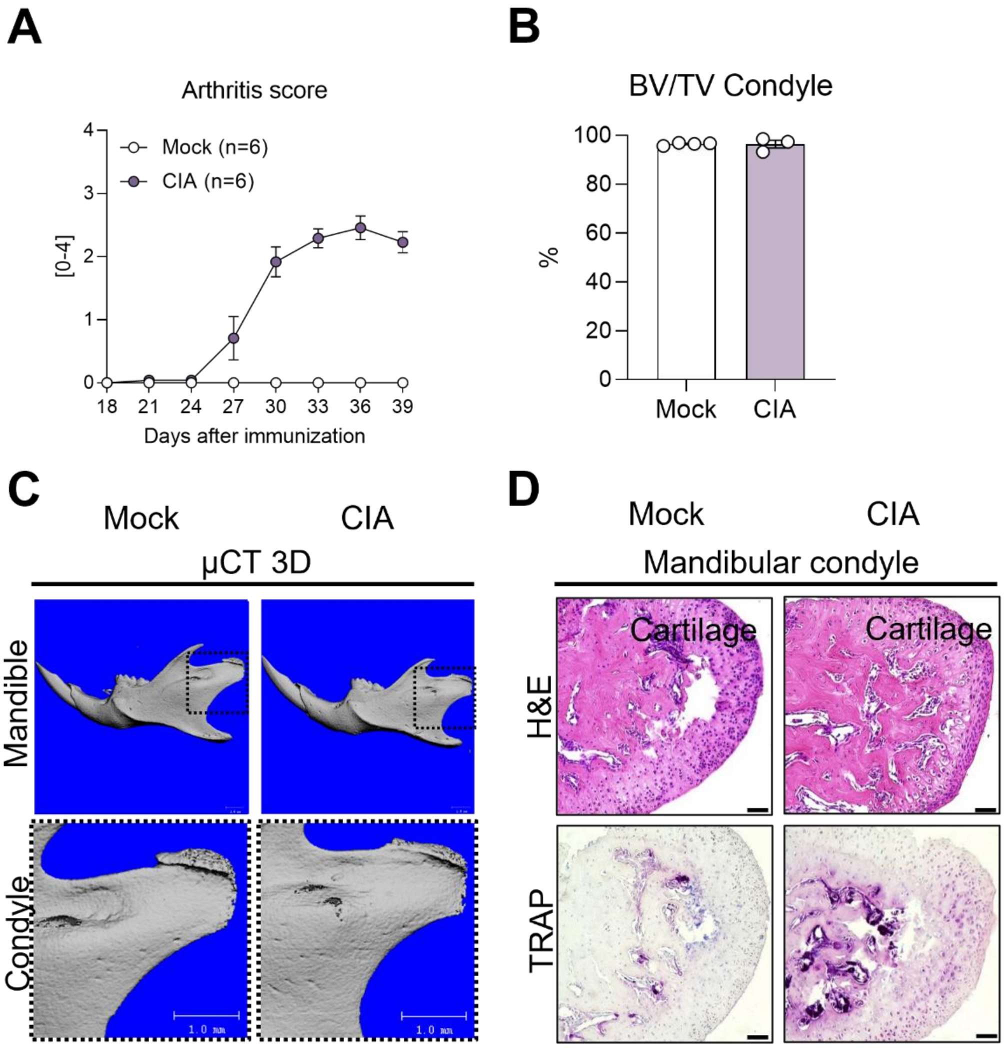

Supplementary Figure 1: The collagen-induced arthritis (CIA) model shows no signs of TMJ involvement. (A) Arthritis progression in the CIA model was assessed using arthritis scores, comparing CIA mice with mock-treated controls (n=6). **(B)** Bone volume per total volume (BV/TV) in the mandibular condyle was measured by micro-computed tomography (µCT) (n=3-4). **(C)** Representative µCT images of the mandibular condyle from CIA mice compared to mock-treated controls. The scale bar indicates 1 mm. **(D)** Hematoxylin and eosin (H&E) and tartrate-resistant acid phosphatase (TRAP) staining of the mandibular condyle in CIA mice and mock-treated controls. The scale bar represents 50 µm. Data are presented as mean ± SEM, with symbols representing individual mice.

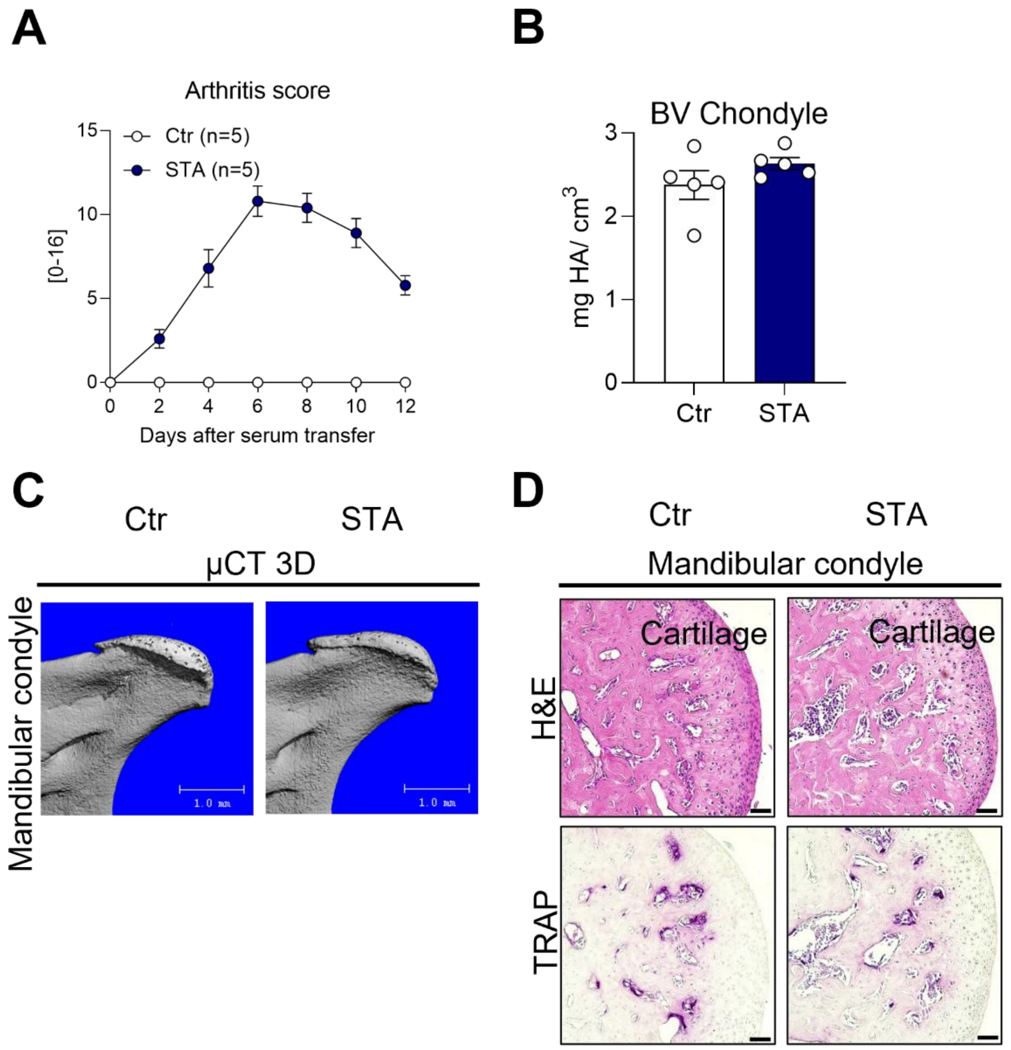

Supplementary Figure 2: The K/BxN serum transfer arthritis (STA) model exhibits no changes in the TMJ. (A) Arthritis progression in the STA model was assessed using arthritis scores, comparing STA mice with mock-treated controls (n=5). **(B)** Bone volume (BV) in the mandibular condyle was measured by µCT (n=5). **(C)** Representative µCT images of the mandibular condyle from STA mice compared to mock-treated controls. The scale bar indicates 1 mm. **(D)** H&E and TRAP staining of the mandibular condyle in STA mice and mock-treated controls. The scale bar represents 50 µm. Data are presented as mean ± SEM, with symbols representing individual mice.

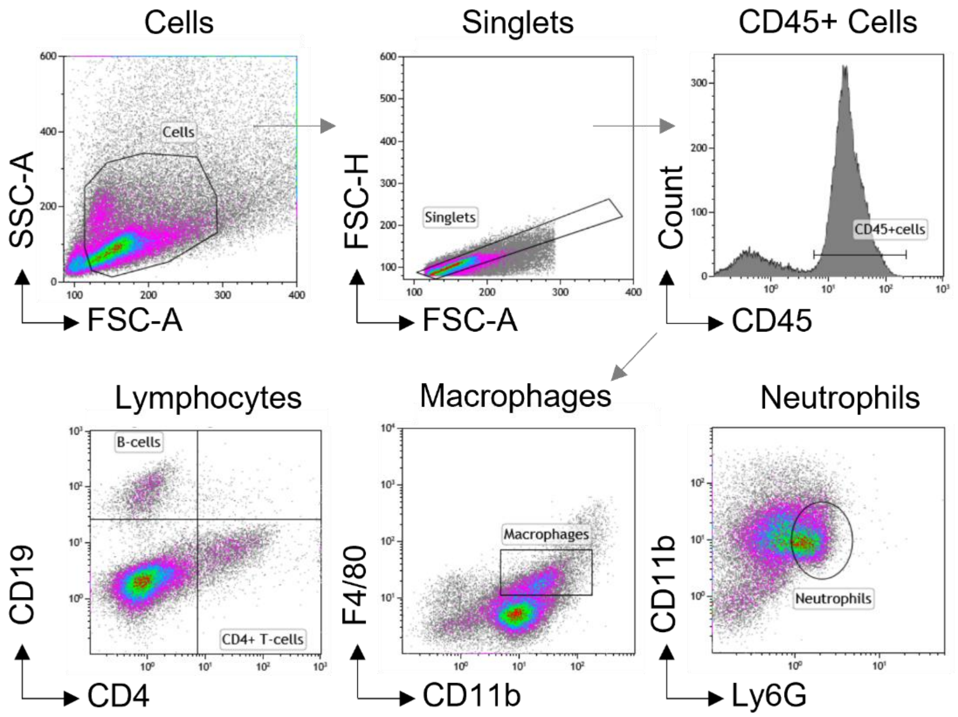

Supplementary Figure 3: Gating strategy for identifying immune cell populations. The analysis included $CD45^+$ immune cells, $CD4^+$ T helper cells, $CD19^+$ B cells, $CD11b^+F4/80^+$ macrophages, and $CD11b^+Ly6G^+$ neutrophils.

A

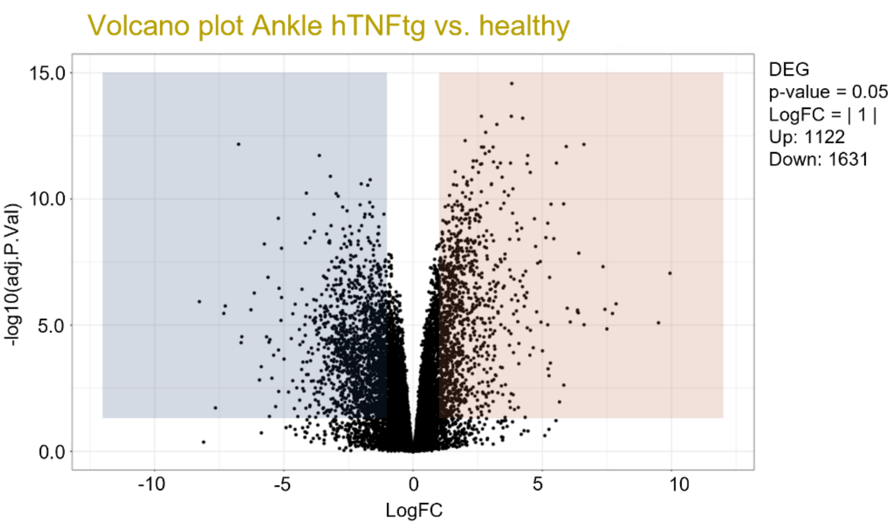

B

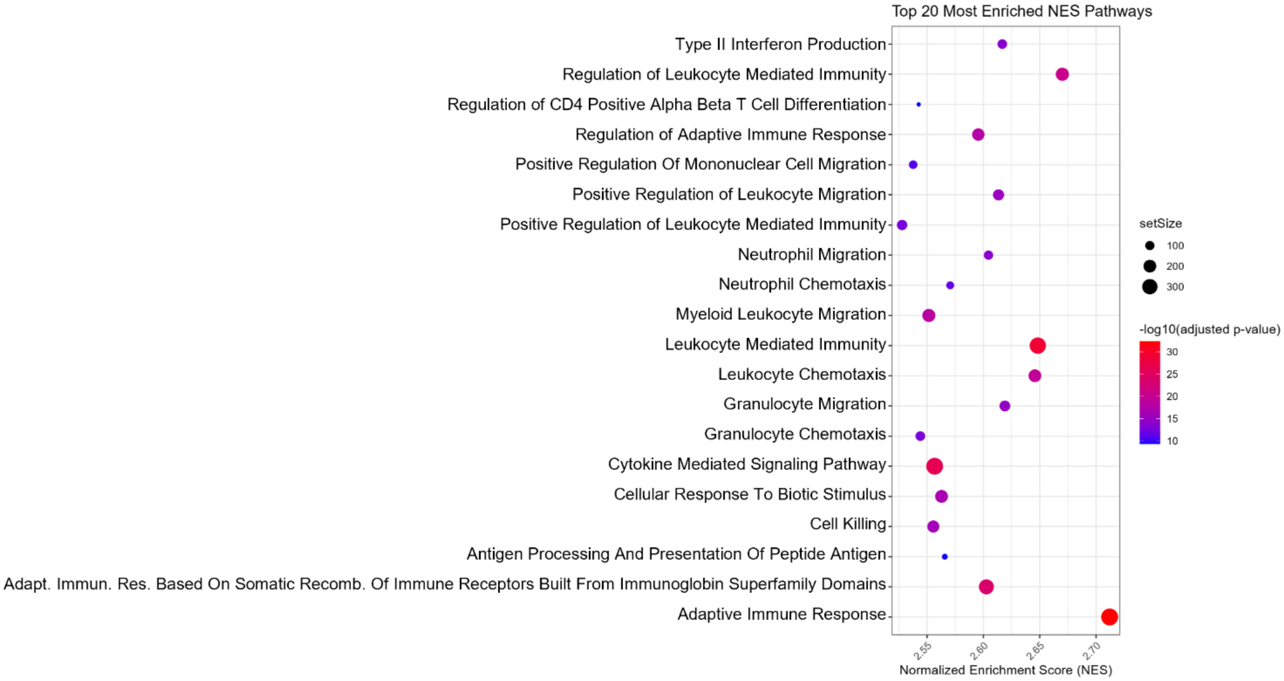

Supplementary Figure 4: Bulk RNA sequencing reveals significant changes in the expressional profile of the arthritic ankle joint compared to healthy conditions. (A) Volcano plot depicting differentially expressed genes (DEGs) between Ankle hTNFtg and Ankle Control groups. **(B)** Gene Ontology (GO) enrichment bubble plot comparing Ankle hTNFtg and Ankle Control groups. Differential expression analysis was conducted with a log2 fold change threshold of 1.0. *P*-values were adjusted using the Benjamini–Hochberg method to control the false discovery rate (FDR). Genes with an adjusted *P*-value (*Padj*) less than 0.05 were considered differentially expressed.

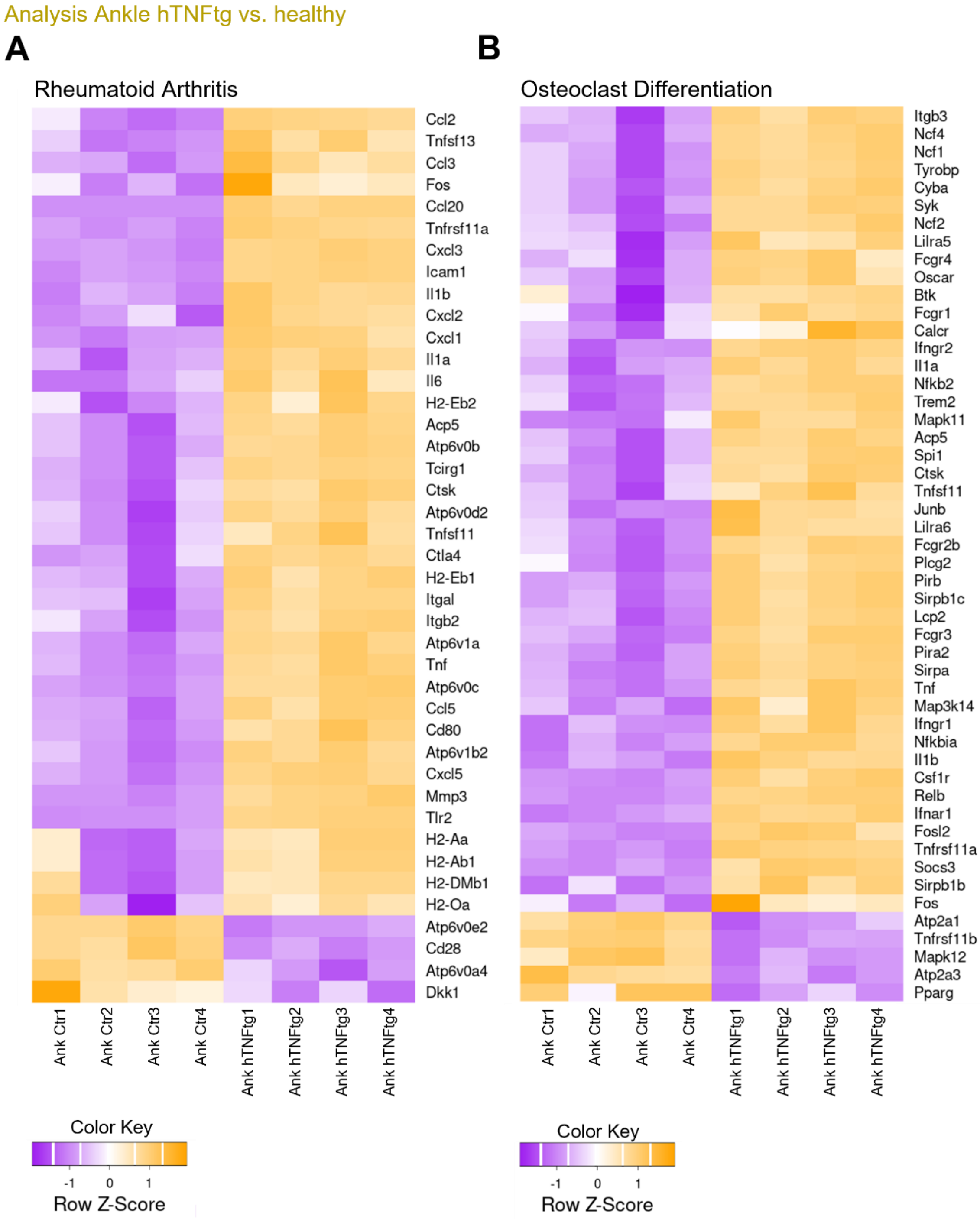

Supplementary Figure 5: Heatmaps presenting increased expression of rheumatoid arthritis and osteoclast differentiation-associated genes in the arthritic ankle joint compared to healthy conditions. (A) Heatmap illustrating rheumatoid arthritis-related differentially expressed genes (DEGs) between Ankle hTNFtg and Ankle Control groups. **(B)** Heatmap showing osteoclast differentiation-related DEGs between Ankle hTNFtg and Ankle Control groups. Differential expression analysis was conducted with a log2 fold change threshold of 1.0. *P*-values were adjusted using the Benjamini–Hochberg method to control the false discovery rate (FDR). Genes with an adjusted *P*-value (*Padj*) less than 0.05 were considered differentially expressed.

Analysis Ankle hTNFtg vs. healthy

A

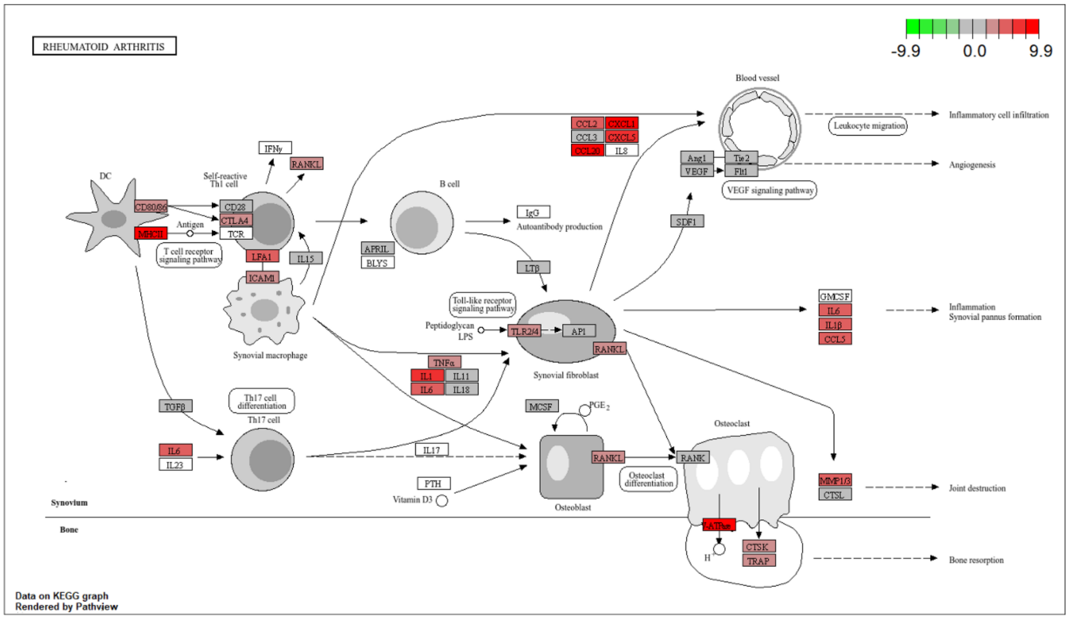

B

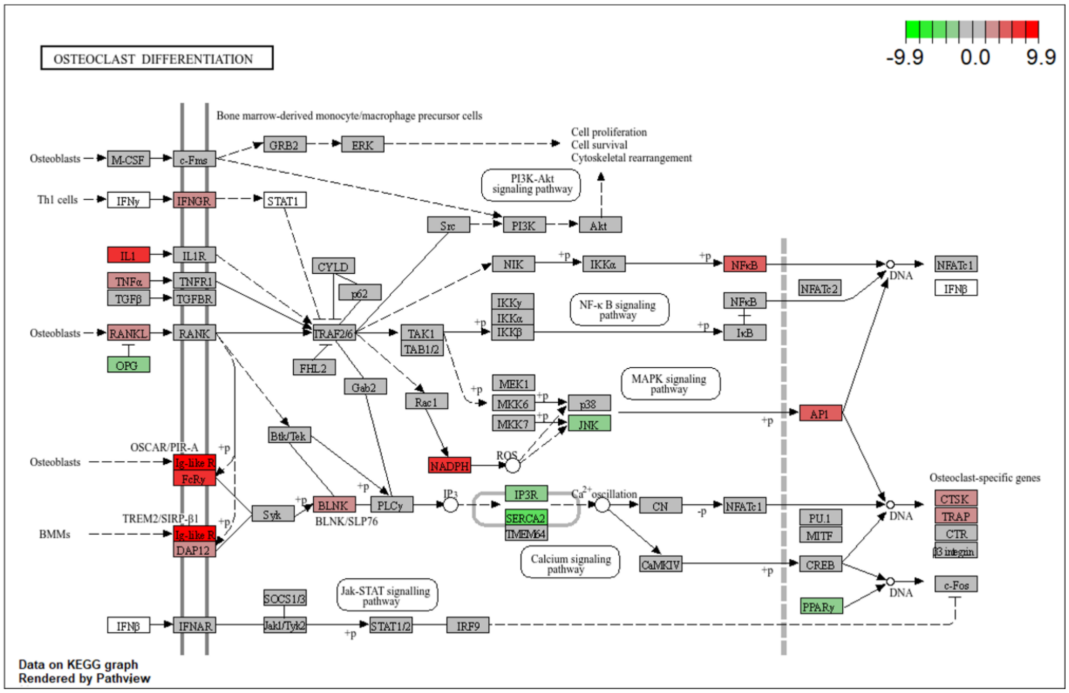

Supplementary Figure 6: KEGG pathways showing enhanced expression of rheumatoid arthritis and osteoclast differentiation-associated genes in the arthritic ankle joint compared to healthy conditions. (A) KEGG pathway showing key rheumatoid arthritis-related differentially expressed genes (DEGs) between Ankle hTNFtg and Ankle Control groups. **(B)** KEGG pathway presenting key osteoclast differentiation-related DEGs between Ankle hTNFtg and Ankle Control groups. Differential expression analysis was conducted with a log2 fold change threshold of 1.0. *P*-values were adjusted using the Benjamini–Hochberg method to control the false discovery rate (FDR). Genes with an adjusted *P*-value (*Padj*) less than 0.05 were considered differentially expressed.

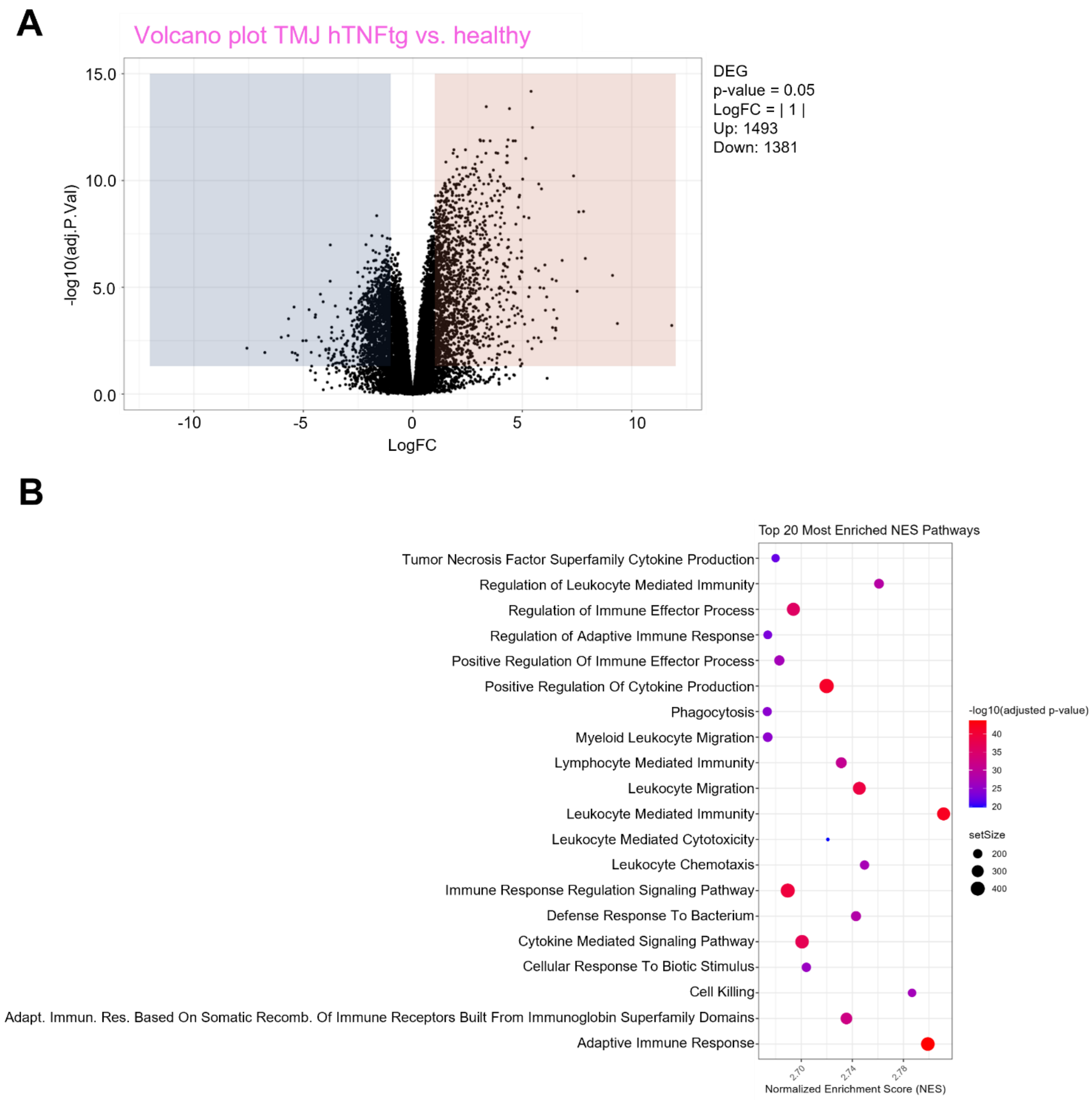

Supplementary Figure 7: Bulk RNA sequencing reveals significant changes in the expressional profile of the TMJ under arthritic conditions compared to healthy conditions. (A) Volcano plot depicting differentially expressed genes (DEGs) between TMJ hTNFtg and TMJ Control groups. **(B)** Gene Ontology (GO) enrichment bubble plot comparing TMJ hTNFtg and TMJ Control groups. Differential expression analysis was conducted with a log2 fold change threshold of 1.0. *P*-values were adjusted using the Benjamini–Hochberg method to control the false discovery rate (FDR). Genes with an adjusted *P*-value (*Padj*) less than 0.05 were considered differentially expressed.

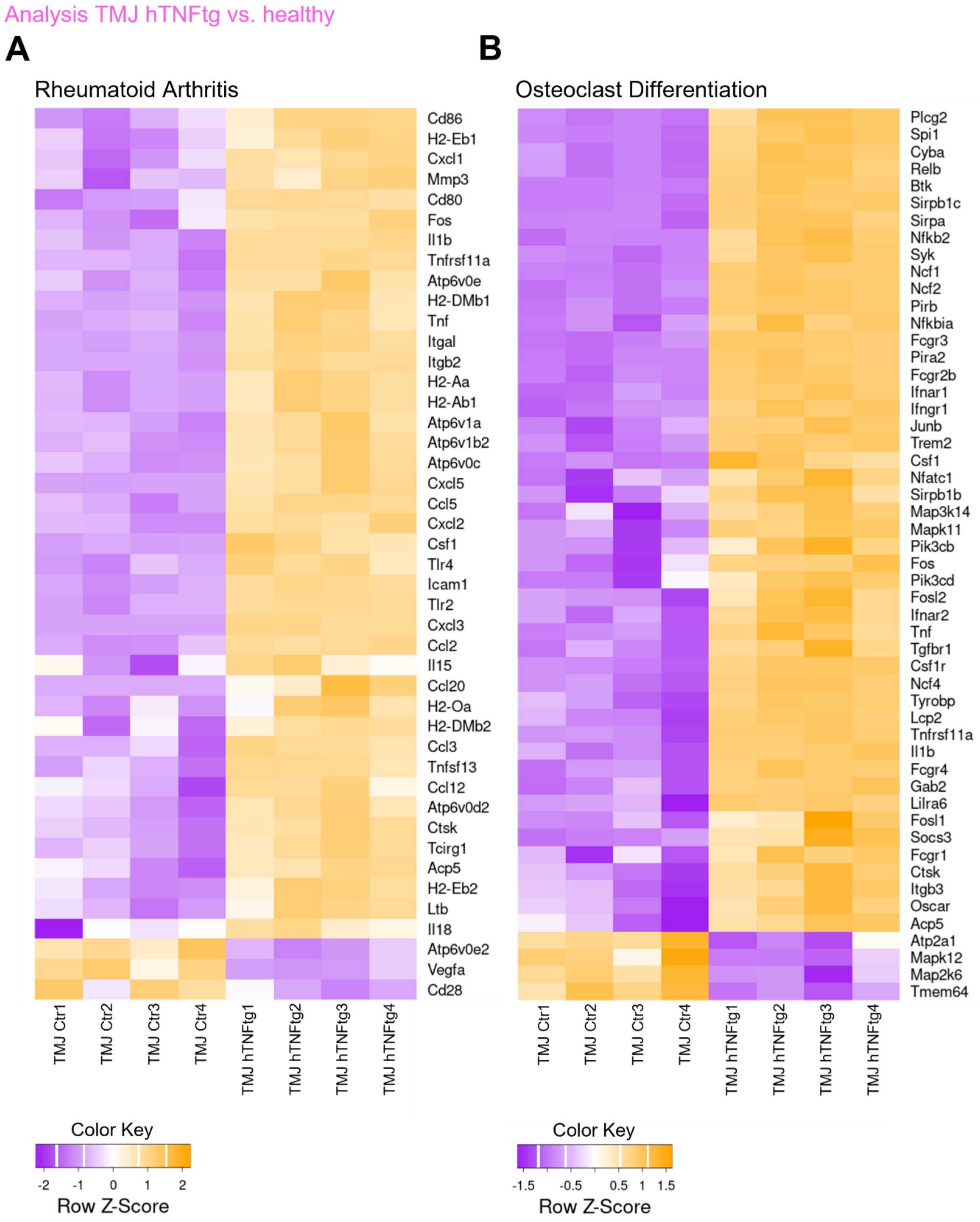

Supplementary Figure 8: Heatmaps depicting enhanced expression of rheumatoid arthritis and osteoclast differentiation-associated genes in the hTNFtg TMJ compared to healthy conditions. **(A)** Heatmap demonstrating rheumatoid arthritis-related differentially expressed genes (DEGs) between TMJ hTNFtg and TMJ Control groups. **(B)** Heatmap presenting osteoclast differentiation-related DEGs between TMJ hTNFtg and TMJ Control groups. Differential expression analysis was conducted with a log2 fold change threshold of 1.0. *P*-values were adjusted using the Benjamini–Hochberg method to control the false discovery rate (FDR). Genes with an adjusted *P*-value (*Padj*) less than 0.05 were considered differentially expressed.

Analysis TMJ hTNFtg vs. healthy

A

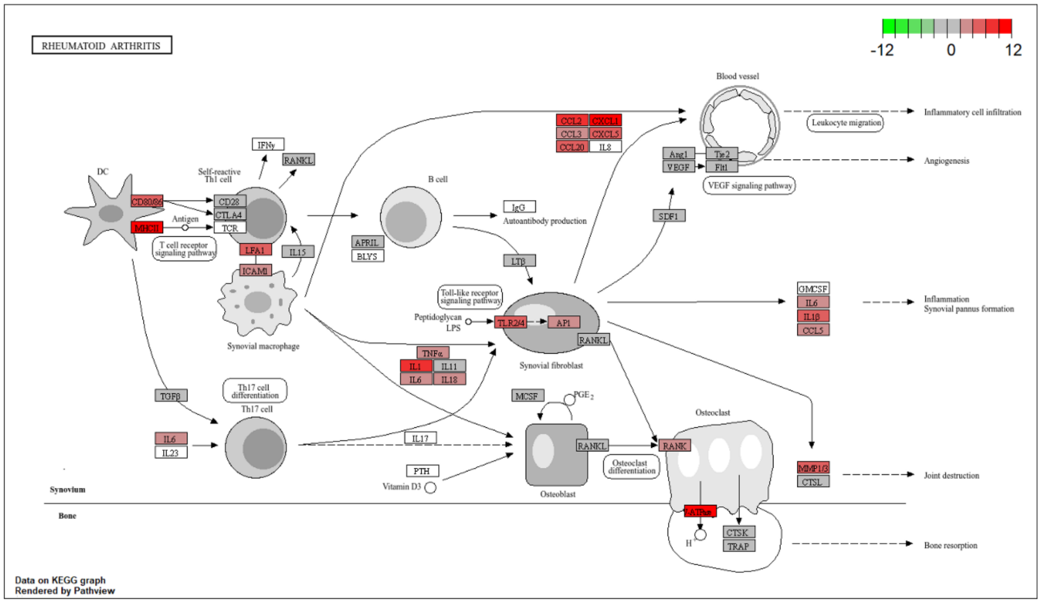

B

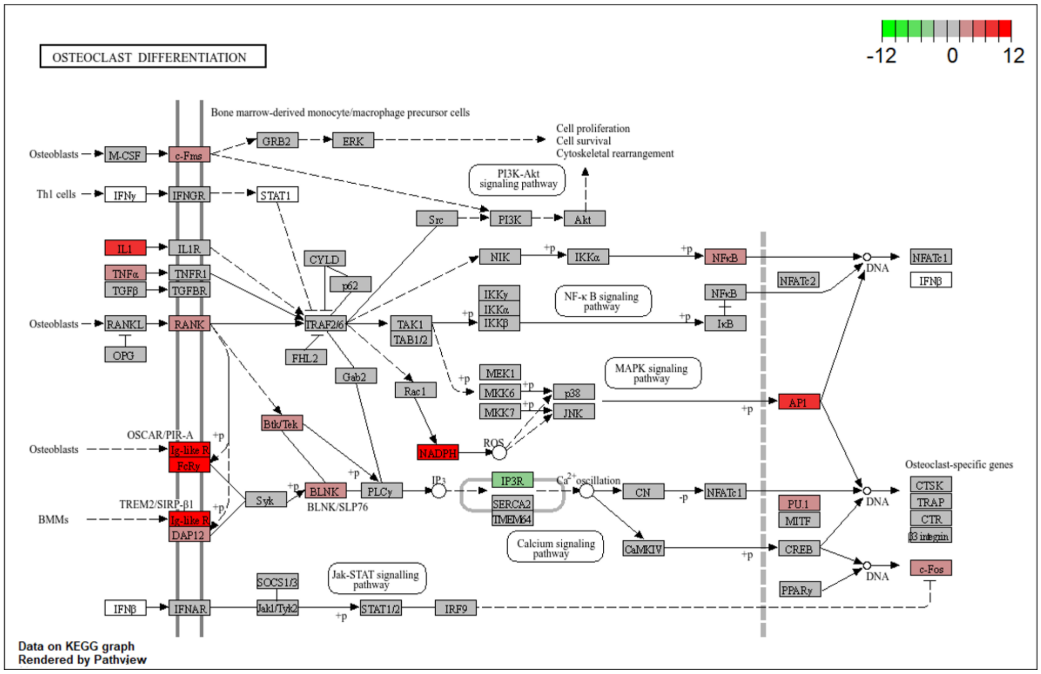

Supplementary Figure 9: KEGG pathways presenting the upregulation of rheumatoid arthritis and osteoclast differentiation-associated genes in the hTNFtg TMJ compared to healthy conditions. (A) KEGG pathway showing key rheumatoid arthritis-related differentially expressed genes (DEGs) between TMJ hTNFtg and TMJ Control groups. **(B)** KEGG pathway presenting key osteoclast differentiation-related DEGs between TMJ hTNFtg and TMJ Control groups. Differential expression analysis was conducted with a log2 fold change threshold of 1.0. *P*-values were adjusted using the Benjamini–Hochberg method to control the false discovery rate (FDR). Genes with an adjusted *P*-value (*Padj*) less than 0.05 were considered differentially expressed.

3 Supplementary References

[1] D.D. Brand, K.A. Latham, and E.F. Rosloniec, Collagen-induced arthritis. Nat Protoc 2 (2007) 1269-75.

[2] V. Kouskoff, A.S. Korganow, V. Duchatelle, C. Degott, C. Benoist, and D. Mathis, Organ-specific disease provoked by systemic autoimmunity. Cell 87 (1996) 811-22.

[3] D. Andreev, K. Kachler, M. Liu, Z. Chen, B. Krishnacoumar, M. Ringer, S. Frey, G. Kronke, D. Voehringer, G. Schett, and A. Bozec, Eosinophils preserve bone homeostasis by inhibiting excessive osteoclast formation and activity via eosinophil peroxidase. Nat Commun 15 (2024) 1067.
